# *Pseudomonas aeruginosa* manipulates redox and iron homeostasis of its microbiota partner *Aspergillus fumigatus* via phenazines

**DOI:** 10.1038/srep08220

**Published:** 2015-02-10

**Authors:** Benoit Briard, Perrine Bomme, Beatrix E. Lechner, Gaëtan L. A. Mislin, Virginie Lair, Marie-Christine Prévost, Jean-Paul Latgé, Hubertus Haas, Anne Beauvais

**Affiliations:** 1 Unité des Aspergillus Institut Pasteur, Paris, France; 2 Université Paris Diderot, Sorbonne Paris Cité, Cellule Pasteur, Paris, France; 3 Plateforme de microscopie ultrastructurale, Institut Pasteur, Paris, France; 4 Biocenter-Division of Molecular Biology, Innsbruck Medical University, Innsbruck, Austria; 5 UMR 7242 Biotechnologie et Signalisation Cellulaire, Université de Strasbourg-CNRS, France; 6 PSL Research University, Chimie ParisTech-CNRS, Institut de Recherche de Chimie Paris, 75005, Paris, France

## Abstract

The opportunistic fungal pathogen *Aspergillus fumigatus* is increasingly found as a coinfecting agent along with *Pseudomonas aeruginosa* in cystic fibrosis patients. Amongst the numerous molecules secreted by *P. aeruginosa* during its growth, phenazines constitute a major class. *P. aeruginosa* usually secreted four phenazines, pyocyanin (PYO), phenazine-1-carboxamide (PCN), 1-hydroxyphenazine (1-HP) and phenazine-1-carboxylic acid (PCA). These phenazines inhibited the growth of *A. fumigatus* but the underlying mechanisms and the impact of these four phenazines on *A. fumigatus* biology were not known. In the present study, we analyzed the functions of the four phenazines and their mode of action on *A. fumigatus*. All four phenazines showed *A. fumigatus* growth inhibitory effects by inducing production of reactive oxygen species (ROS), specifically O_2_^**·**−^, and reactive nitrogen species (RNS), ONOO^−^. *A. fumigatus* Sod2p was the major factor involved in resistance against the ROS and RNS induced by phenazines. Sub-inhibitory concentrations of PYO, PCA and PCN promote *A. fumigatus* growth by an independent iron-uptake acquisition. Of the four phenazines 1-HP had a redox-independent function; being able to chelate metal ions 1-HP induced *A. fumigatus* iron starvation. Our data show the fine-interactions existing between *A. fumigatus* and *P. aeruginosa*, which can lead to stimulatory or antagonistic effects.

P*seudomonas aeruginosa* is the most common cause of bacterial infections in cystic fibrosis patients. Amongst the numerous molecules secreted by *P. aeruginosa* during its growth, phenazines constitute a major class. Phenazines are small diffusible quorum sensing molecules which penetrate easily all kind of cells^1^. These natural pigments comprise blue for pyocyanin (PYO), yellow for phenazine-1-carboxylic acid (PCA) and phenazine-1-carboxamide (PCN) and orange for 1-hydroxyphenazine (1-HP). They are considered as the main virulence factor of *P. aeruginosa* against a broad range of target organisms, including other bacteria, fungi and mammalian cells^1^,^2^,^3^. In cystic fibrosis sputum, phenazines are present at concentrations in the range of 1 to 100 μM^4^, and their concentration increases with a concomitant decline in lung function. In these patients, *P. aeruginosa* forms a biofilm in which a hypoxic gradient is generated due to the overproduction of alginate, creating an anaerobic environment which increases phenazine toxicity^5^. To date, four main phenazines have been identified in *P. aeruginosa*: Phenazine-1-carboxylic acid (Phenazine-1-carboxylate, PCA), which is produced from chorismic acid. PCA is further modified in pyocyanin (PYO), 1-hydroxyphenazine (1-HP), or phenazine-1-carboxamide (PCN) (Supplementary Figure 1). Phenazines are endogenous redox-active molecules and it has been demonstrated that this activity promotes *P. aeruginosa* growth and also survival under iron limiting conditions, as in cystic fibrosis.

The function and toxicity of phenazines produced by *P. aeruginosa* have been mainly investigated *in vitro* by using PYO with epithelial bronchial host cells, the fungus *Candida albicans* or the nematode *Caenorhabditis elegans*^6^. PYO is known to induce oxidative stress, mainly in the form of O_2_^**·**−^ radicals in eukaryotic and prokaryotic cells^7^,^8^. PYO reaches the mitochondria, where it enhances the generation of reactive oxidant species. This induced oxidative burst is toxic for the targeted cells and mediates killing of the lung epithelial cells or other opportunistic bacteria or fungi, which compete for the same niches. PYO enhances extracellular DNA release by *P. aeruginosa* via H_2_O_2_ generation^9^. Extracellular DNA is then used by the bacteria to improve the maturation and structural integrity of its biofilm, thereby enhancing the antibacterial resistance^10^. PYO and PCA can accept electrons from the respiratory chain through a complex III-dependent process in the mitochondria, which potentially decreases the cellular ATP content^11^,^12^. In addition to their role in oxide reduction process, PYO and PCA promote *P. aeruginosa* biofilm development via ferrous iron acquisition from iron-containing host proteins like transferrin or haemoglobin^13^. The stimulation of Fe(II) acquisition may also favor the behavior of other infectious agents present in the upper respiratory tract of cystic fibrosis patients. However, nothing is known until now about the mode of action of the other phenazines, PCN and 1-HP.

*A. fumigatus* is isolated in 60% of the cystic fibrosis patients with *P. aeruginosa* infection^14^, demonstrating a close relationship between the established colonization by *P. aeruginosa* and a superinfection by *A. fumigatus*. While numerous studies have investigated *P. aeruginosa/C. albicans* interactions, interactions between *P. aeruginosa* and *A. fumigatus* remain poorly understood despite yearly increases in this coinfection but with the bacterial/yeast infections remaining stable^15^. *A. fumigatus* and *P. aeruginosa* share common features, such as the adhesion to basal membrane, chronic colonization of the upper respiratory track, induction of inflammation, causing damage to the respiratory functions of the patients. Previous studies demonstrated that phenazines inhibited growth of *A. fumigatus* but the underlying mechanisms were not characterized^16^,^17^. Moreover, nothing is known about the impact of these four phenazines on *A. fumigatus* biology apart from the fact that 1-HP induces production of siderophores by this fungus. Based on the functions of *P. aeruginosa* on host cells, it was hypothesized that these phenazines could be inhibitory to *A. fumigatus* growth due to ROS stimulation and perturbation in the availability of iron.

In *A. fumigatus*, different ROS scavengers have been identified in the genome including three superoxide-dismutases (Sod1p, 2p, 3p) and three catalases (CatAp, Cat1p, 2p)^18^,^19^. Sod1p and Sod3p are cytoplasmic, whereas Sod2p is mitochondrial. Δ*sod1* and Δ*sod2* single mutants and the triple Δ*sod1*/Δ*sod2*/Δ*sod3* mutant are characterized by an increased sensitivity to menadione, which leads to superoxide anions (O_2_^**·**−^). CatAp is expressed in the conidia whereas Cat1p and Cat2p are found in the mycelium. The double Δ*cat1*Δ*cat2* mutant with no catalase activity in the mycelium exhibited only slightly increased sensitivity to H_2_O_2_^19^. In *A. fumigatus*, two transcription factors are specifically induced by oxidative stress, Skn7p and Yap1p^20^,^21^. Expression of *SKN7* was specifically induced by H_2_O_2_ and not menadione, leading to the formation of peroxide (O_2_^2−^)^20^. *YAP1*-deficiency increases the susceptibility of *A. fumigatus* to both H_2_O_2_ and menadione.

*A. fumigatus* produces two extracellular siderophores for iron acquisition, i.e., fusarinine C (FsC) and triacetylfusarinine C (TAFC), and two intracellular siderophores for storage and transport of iron, i.e, ferricrocin (FC) and hydroxyferricrocin (HFC)^22^. Extracellular siderophores are essential for fungal growth in the host because they help to acquire iron from transferrin during the course of infection^23^,^24^. The siderophore biosynthetic pathway is schematized in Supplementary Figure 2. SidAp catalyzes the first committed step in biosynthesis of extra- and intracellular siderophores and consequently, the Δ*sidA* mutant is unable to produce any siderophores^25^. The subsequent, pathways for downstream synthesis of extra- and intracellular siderophores split, with SidFp and SidDp are required for production of the extracellular siderophores while SidCp is required for production of intracellular siderophores^22^. The expression of these genes is alternately regulated by two transcription factors, SreAp and HapXp. *SREA* is expressed in presence of iron repressing the synthesis of siderophores and alternative reductive iron assimilation. During iron starvation conditions however, *HAPX* is expressed, activating the synthesis of siderophores and simultaneously repressing iron-consuming pathways^26^,^27^,^28^.

In this study, we analyzed the functions of the four phenazines produced by *P. aeruginosa* and their mode of action on growth and survival of *A. fumigatus*. For the first time we demonstrate that all phenazines have an inhibitory effect by inducing production of reactive oxygen species (ROS), specifically O_2_^**·**−^, and reactive nitrogen species (RNS), ONOO^−^. We found that *A. fumigatus* Sod2p was the major factor involved in resistance against the ROS and RNS induced by phenazines. We also demonstrate that subinhibitory concentrations PYO, PCA and PCN can promote *A. fumigatus* growth by an independent iron-uptake acquisition. Furthermore, we show for the first time that 1-HP has a redox-independent function, as being able to chelate metal ions and to induce iron starvation in *A. fumigatus*.

## Results

### Phenazines modify the morphology of *A. fumigatus* hyphae and mitochondria

**Figure 1:**
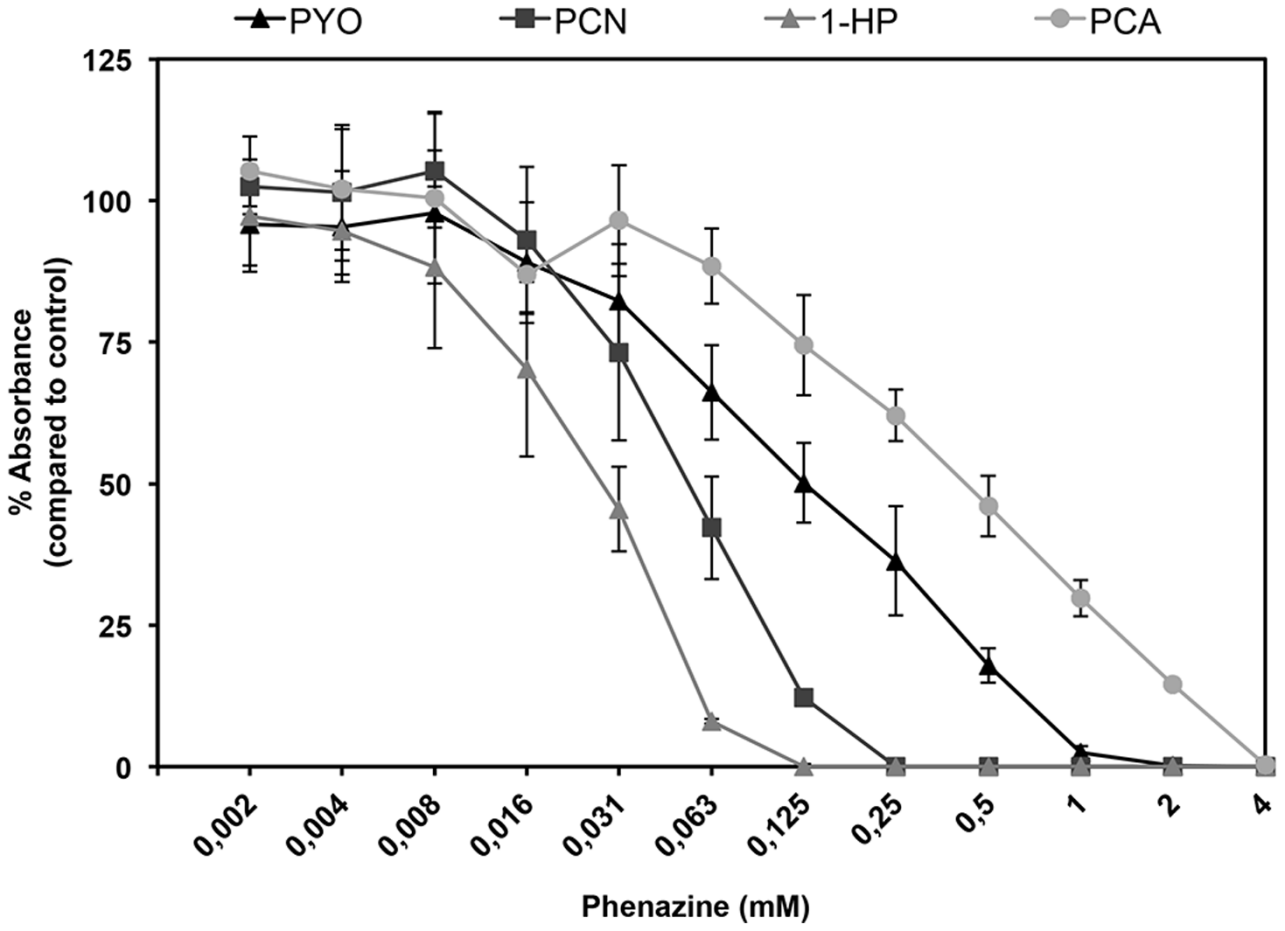
MIC of phenazines against *A. fumigatus.* Growth of *A. fumigatus* CEA17ΔakuB^KU80^ (WT) was determined in the presence of increasing concentrations of the four phenazines (PYO, PCN, 1-HP and PCA). Mycelial growth was estimated by reading absorbance at 560 nm using the crystal violet method after 20 h in 2YT at 37°C, and compared to the control growing in absence of phenazine and normalized at 100% absorbance at 560 nm.

**Figure 2:**
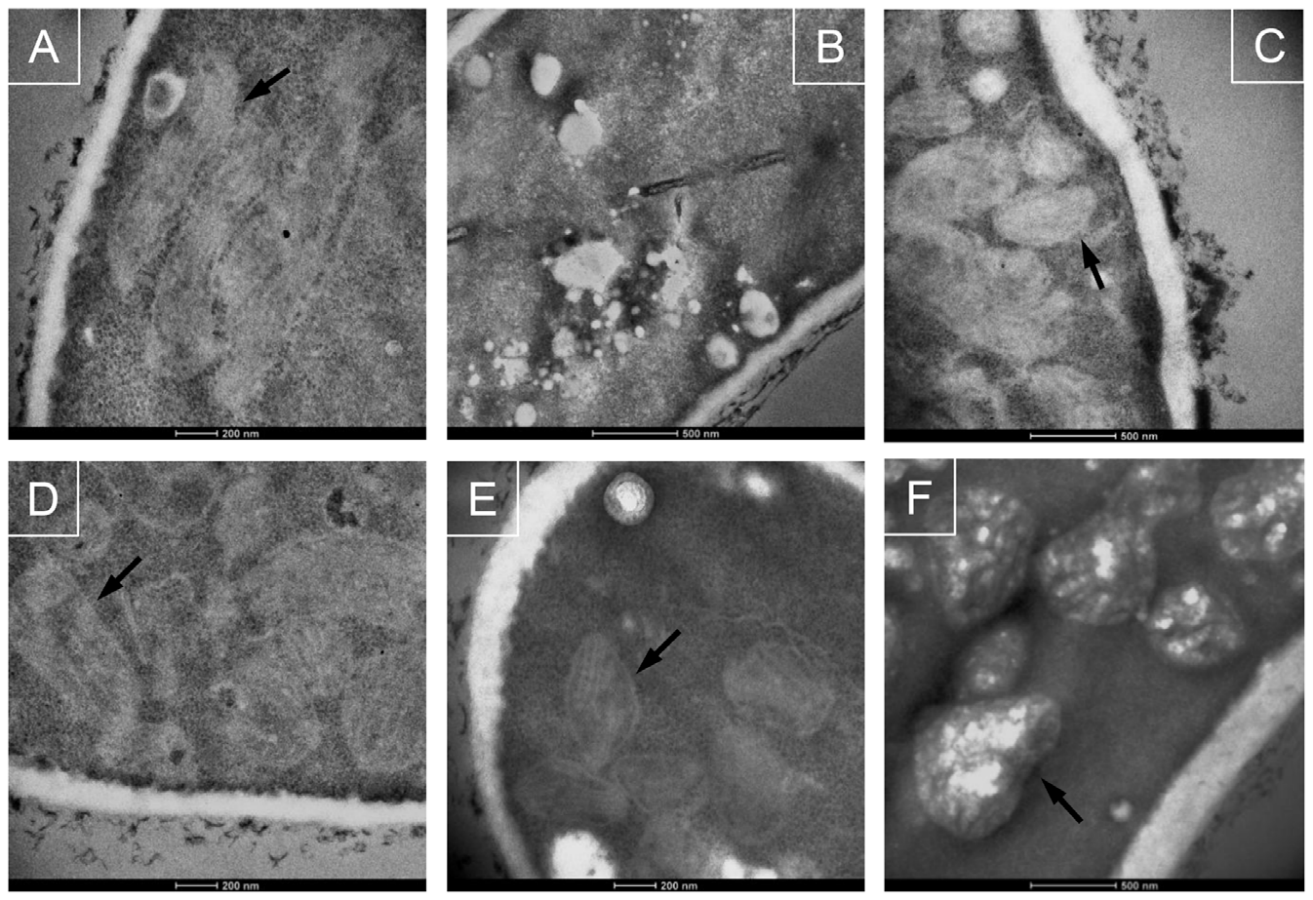
Ultrastructural morphology of *A. fumigatus* hyphae in the presence of phenazines. (A) *A. fumigatus* conidia incubated for 20 h growth at 30°C in the presence of 1% DMSO. (B–F) *A. fumigatus* conidia incubated for 18 h growth at 37°C in the presence of 1 mM PYO (B, C), 125 μM PCN (D), 62.5 μM 1-HP (E) or 2 mM PCA (F). Arrows show the mitochondria in *A. fumigatus* cells.

We first set out to determine the inhibitory effects of phenazines on the growth of *A. fumigatus*. As shown in figure 1, all phenazines tested exhibited differing growth inhibitory activities. PCA was the least active phenazine against *A. fumigatus* with a minimal inhibitory concentration (MIC) of 4 mM (Figure 1). This could be a result of charge repulsions between both negatively charged PCA and hyphal cells leading to decreased penetration^29^,^30^. To verify this hypothesis, we performed the same experiment with PCA at pH 5 which is close to the previously defined pKa of 4.25^31^. At this pH, PCA has neutral charges. Since pH 4.25 is very detrimental for the growth of *A. fumigatus*, we tested if the MIC of PCA at pH 5 was modified due to an increased penetration of the molecule through the cell. Effectively, PCA MIC dropped to 0.5 mM at pH 5 (Supplementary Table 1). At pH 7, PCN and 1-HP were the most active with an MICs of 0.25–0.5 mM and 0.125–0.25 mM, respectively, while PYO presented an intermediate inhibitory activity amongst the four phenazines tested, with a MIC of 2 mM (Figure 1). At pH 5, the MICs of PYO, PCN and 1-HP decreased only by a factor of 2 (Supplementary Table 1). When grown in the absence of phenazines, *A. fumigatus* conidia started swelling after 4 h at 37°C. After 10 h at 37°C, or 20 h at 30°C, all conidia had germinated and presented long branched hyphae (Supplementary Figure 3A and B). In the presence of MIC_50_ concentrations of phenazines, swelling and germination were delayed significantly and observed only after 18 h at 37°C. Moreover, the rate of germination was very heterogeneous, with swollen conidia, small germ tubes and hyphae observed between 18 to 20 h at 37°C. The branching of the hyphae which reflects the initial stage of mycelial network development was reduced in the presence of 1-HP and PYO, (Supplementary Figure 3D, E) and totally abolished in presence of PCN (Supplementary Figure 3F). In contrast, no difference in branching was seen between control and PCA treated cultures (Supplementary Figure 3C). Transmission electron microscopic observations showed that the hyphal cell wall was not modified in presence of phenazines (Figure 2). However, mitochondrial morphology was altered as many appeared round (arrow, Figure 2C–F).

### Phenazines have redox activity on *A. fumigatus* swollen conidia and hyphae

Incubating 14 h-old hyphae with phenazines for 6 h at 37°C resulted in mycelium being stained with the natural pigment characteristic of each phenazine, i.e., in greenish-brown in the presence of PYO, yellow with PCN and 1-HP or yellow-pink in the presence of PCA (data not shown).

**Figure 3:**
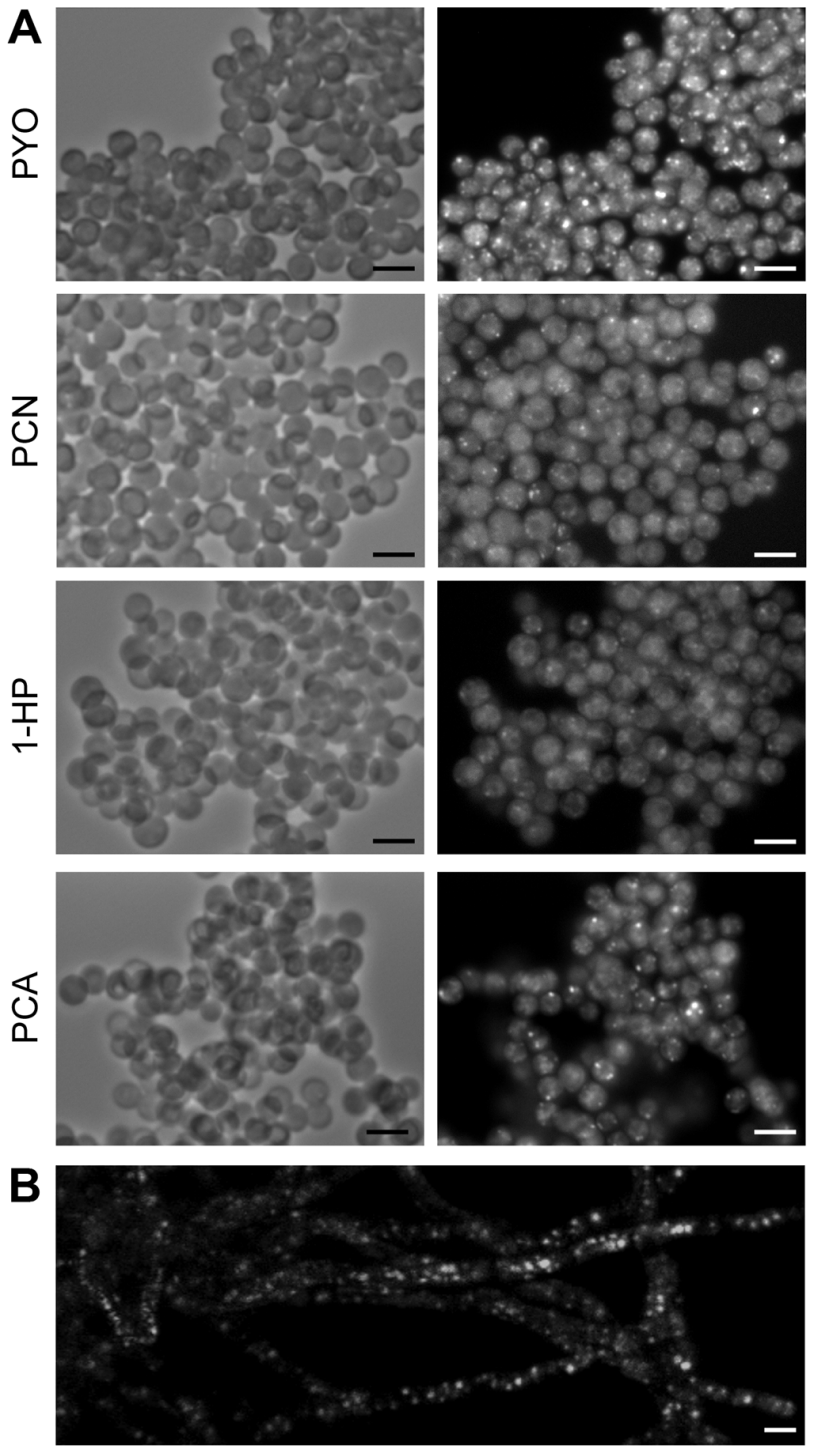
Phenazines penetrate and have redox activity. (A) Swollen conidia incubated with phenazines (PYO, PCN, 1-HP, PCA) in 2YT at MIC concentrations for 1 h and observed under emission-specific wavelengths. (B) Mycelium was incubated in 2YT 1 h with PYO (MIC concentration). Scale bar represents 5 μm.

In addition to being pigments, phenazines also possess fluorescence capabilities. While all have excitation wavelengths falling within the UV spectrum, the emission wavelength is specific to each of them: 329/492 nm for PYO, 395/566 nm for PCA and PCN and 355/485 nm for 1-HP. PCA, 1-HP and PYO were fluorescent only when reduced. Both oxidized and reduced forms of PCN were similarly fluorescent. Consequently, the fluorescence properties of PYO, PCA, PCN and 1-HP permitted us to follow not only their penetration into the *A. fumigatus* cell but also their redox activities. Resting conidia were not fluorescent after incubation with phenazines (data not shown). But swollen conidia (4.5 h in 2YT at 37°C) were fluorescent after 1 h incubation in the presence of PYO, 1-HP, PCN and PCA at MIC concentrations (Figure 3), suggesting that the penetration of the phenazines molecules begins when the conidia start germinating. These results also indicated that these phenazines penetrated into the swollen conidia in their reduced forms. The fluorescence observed with all phenazines in the swollen conidia was diffuse in the cytoplasm but some highly fluorescent spots were observed (Figure 3A). A similar result was observed after 1 h incubation of 14 h-growing hyphae with phenazines at 37°C (Figure 3B and data not shown). Importantly, in the presence of phenazines, mitotracker dye labeling failed to produce any fluorescence (data not shown). Since mitotracker stains only cells harboring active mitochondria, our results suggested that phenazines targeted the mitochondria.

### Phenazines induce reactive oxygen (ROS) and reactive nitrogen (RNS) species

**Figure 4:**
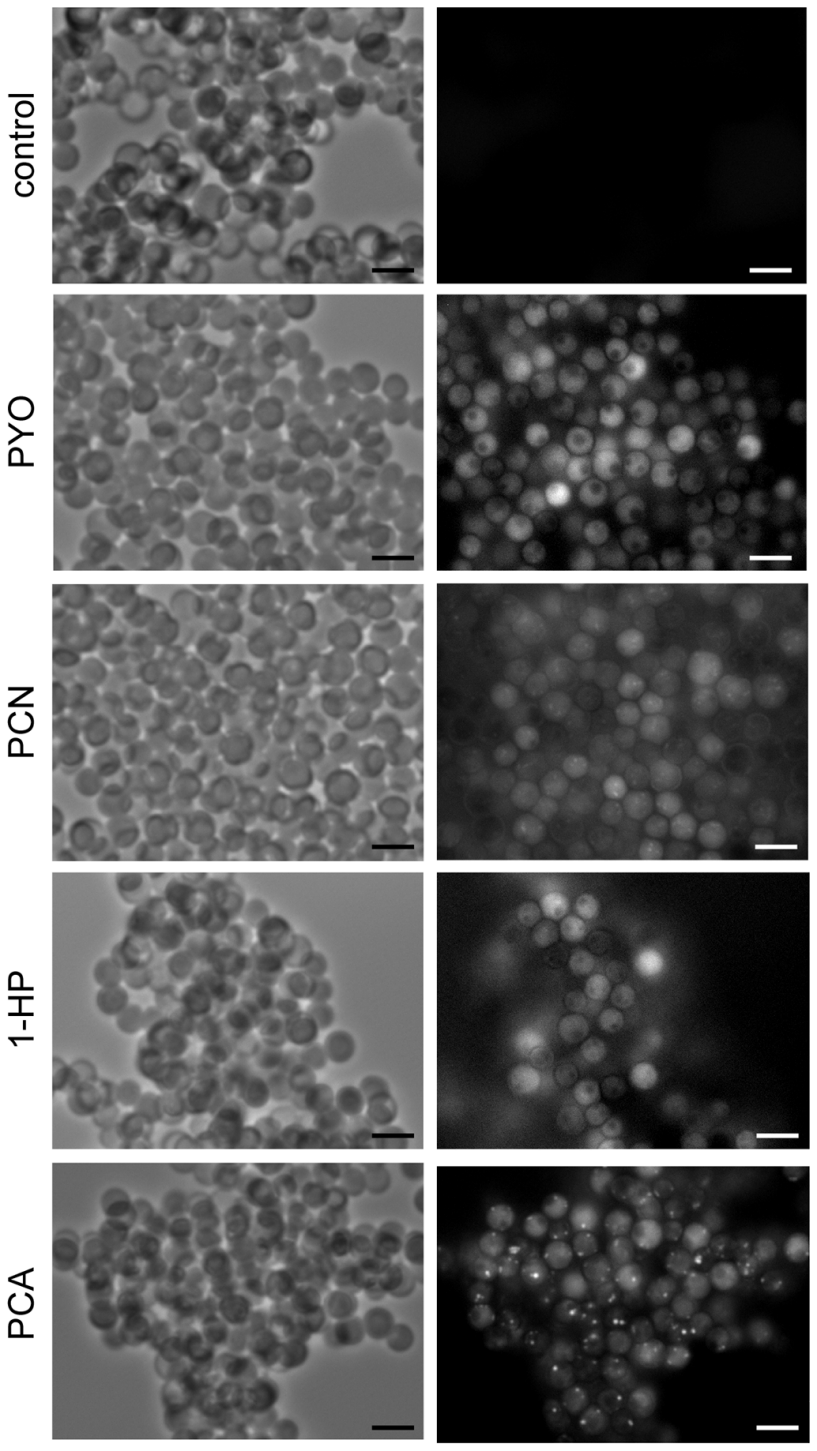
Phenazines induce ROS production in *A. fumigatus* swollen conidia. H_2_DCFDA, a ROS fluorescent probe, was added to swollen conidia prior to the addition of PYO, PCN, 1-HP and PCA at MIC concentrations for 1 h and observed under emission-specific wavelengths. For control swollen conidia were incubated with H_2_DCFDA in absence of phenazines. Scale bar represents 5 μm.

To investigate whether phenazines induced production of ROS in *A. fumigatus*, swollen conidia were incubated for 1 h with phenazines at MIC concentrations after the addition of the non-fluorescent dye H_2_DCFDA, which on oxidized by O_2_^**·**−^ forms the fluorescent molecule DCF. We observed a fluorescence in all phenazines/H_2_DCFDA treated swollen conidia indicating the production of O_2_^**·**−^ radicals (Figure 4). Some fluorescent spots were also observed in the cell, mostly in PCA/H_2_DCFDA treated swollen conidia (Figure 4), suggesting a production of ROS in mitochondria where the phenazines were localized (Figure 3). In order to validate these results, we decided to study *A. fumigatus* mutants deleted in genes involved in the resistance to oxidative stress.

First, we tested the sensitivity to phenazines, of *A. fumigatus* mutants known to be hypersensitive to H_2_O_2_ stress, such as, the transcription factor-deleted mutants Δ*yap1* and Δ*skn7*, and the single and double catalase deleted mutants Δ*cat1*, Δ*cat2*, Δ*catA* and Δ*cat1*Δ*cat2* and Δ*cat1*Δ*catA*, respectively. As shown in table 1, all of the mutants were as sensitive as the parental strain to the four phenazines, showing that ROS detoxification was catalase-independent. Next, we tested the sensitivity of O_2_^**·**−^ hypersensitive superoxide dismutase single deleted, double and triple mutants to phenazines. Although the cytoplasmic superoxide-dismutase deleted mutants, Δ*sod1* and Δ*sod3*, were as susceptible as the parental strain to all phenazines, the mitochondrial superoxide dismutase deletion mutant Δ*sod2* was hypersensitive to all phenazines, particularly to PCA (Table 1). The cytoplasmic superoxide dismutase lacking Δ*sod1*Δ*sod3* mutant showed increased susceptibility only to 1-HP with an MIC of 0.062–0.125 mM compared to 0.125–0.25 mM for the parental strain, but the triple Δ*sod1*Δ*sod3*Δ*sod2* mutant was the most susceptible strain to all phenazines (Table 1). These data suggest that phenazines induce O_2_^**·**−^ formation in the mitochondria and demonstrate that superoxide dismutases, particularly Sod2p, are essential in the protective response of *A. fumigatus* against an increase in intracellular ROS.

**Figure 5:**
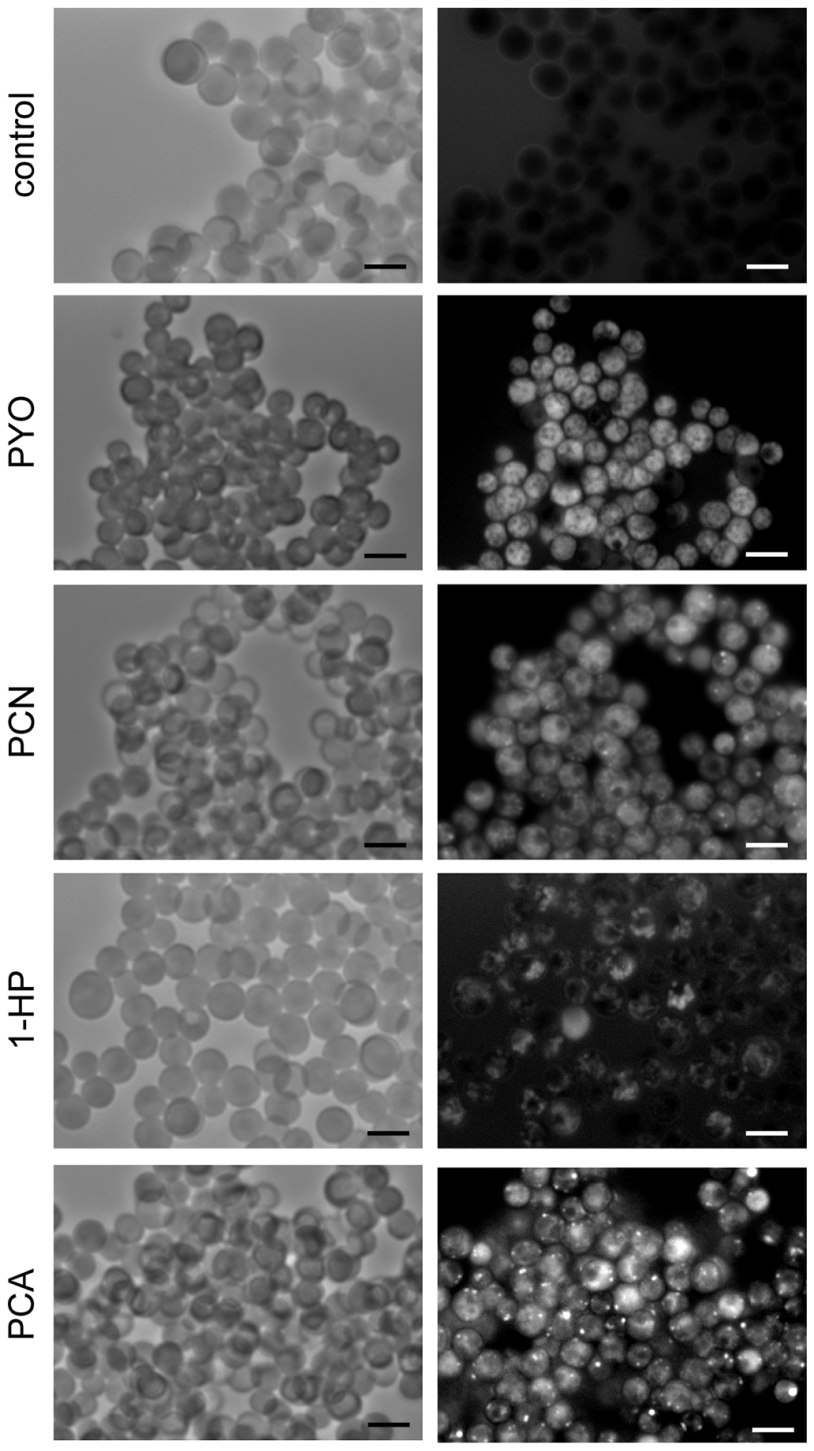
Phenazines induce RNS production in *A. fumigatus* swollen conidia. DHR123, a RNS fluorescent probe, was added to swollen conidia prior to the addition of PYO, PCN, 1-HP and PCA at MIC concentrations for 1 h and observed under emission-specific wavelengths. For control swollen conidia were incubated with DHR123 in absence of phenazines. Scale bar represents 5 μm.

It has been demonstrated previously that an overproduction of O_2_^**·**−^ induces the biosynthesis of the reactive nitrogen species (RNS)^32^. Nitric oxide (NO^**·**^) that is naturally present in mitochondria reacts with O_2_^**·**−^ radicals to produce the highly toxic peroxynitrite radicals (ONOO^−^)^32^. To test the production of ONOO^−^ in *A. fumigatus* cells in presence of phenazines, we incubated swollen conidia for 1 h with phenazines after the addition of dihydrorhodamine 123 (DHR123), a cell-permeable fluorogenic probe used for the detection of ONOO^−^33,34. DHR123 is not fluorescent until oxidized by ONOO^−^ resulting in its fluorescent derivative Rhodamine 123. Swollen conidia were highly fluorescent in presence of phenazines showing that phenazines also induced high production of ONOO^−^ in the fungal cells. Similarly to ROS fluorescence, RNS staining showed diffuse fluorescence in the cytoplasm with some fluorescence spots, especially in PCA/DHR123 treated swollen conidia (Figure 5).

### Phenazines control iron acquisition

#### Impairment iron starvation adaptability increases susceptibility to 1-HP but not to other phenazines

To study the role of phenazines in iron acquisition by *A. fumigatus*, we tested the effect of phenazines on the growth of *A. fumigatus* mutant strains lacking (i) the major iron-regulatory transcription factors, Δ*sreA* and Δ*hapX*, (ii) extracellular siderophore biosynthesis due to deficiency in two enzymes, SidDp and SidFp, or (iii) intracellular siderophores due to deficiency the SidCp enzyme. The MICs of PYO, PCN and PCA were similar between the control strains and mutants (Table 2) suggesting an iron-independent inhibitory effect of these three phenazines. However, while the MIC of 1-HP was similar against Δ*sreA*, Δ*sidC* and the parental strain, the Δ*hapX,* Δ*sidD* and Δ*sidF* mutants, in contrast, were two to four times more susceptible to 1-HP compared to the parental strain (Table 2). Previous studies showed that *HAPX*, *SIDD* and *SIDF* were transcriptionally induced during iron starvation and were required for adaptation under iron-depleted conditions^27^,^28^. In comparison, *SIDC* was less activated during iron-limiting conditions and consequently, Δ*sidC* growth was less affected^27^,^28^. The increased susceptibility of Δ*hapX*, Δ*sidD* and Δ*sidF* mutants to 1-HP suggests that this phenazine causes iron starvation conditions for *A. fumigatus*, possibly by chelation of iron, which is thereby compensated by the activity of HapXp and extracellular siderophore biosynthesis. In agreement with this line of reasoning, supplementation with exogenous purified TAFC restored 1-HP susceptibility of Δ*hapX*, Δ*sidD* and Δ*sidF* mutants to parental strain levels (Table 2).

**Figure 6:**
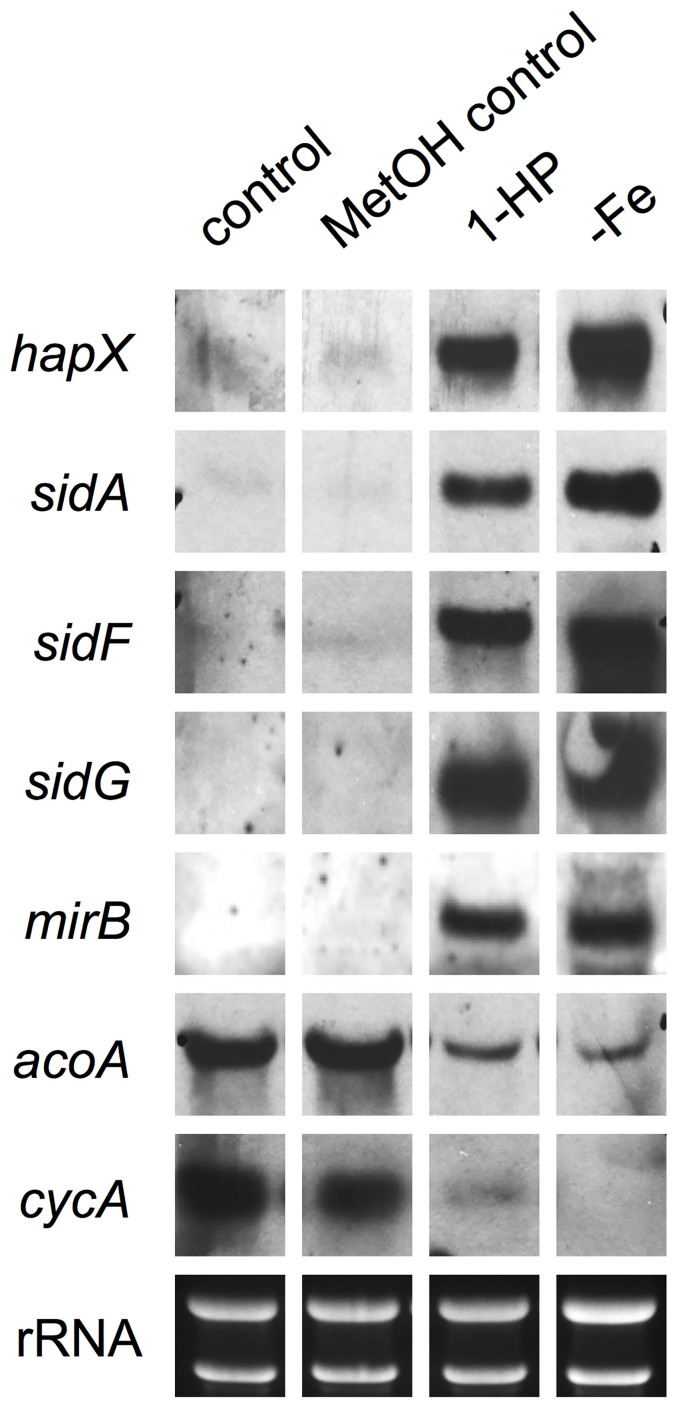
1-HP induces an iron starvation response in *A. fumigatus*. *A. fumigatus* was grown for 12 h in liquid 2YT medium, followed by the addition of 0.0625 mM 1-HP and incubated for an additional 2 h. Mycelia were harvested, total RNA isolated and subjected to Northern blot analysis with the indicated probes. Ribosomal RNA (rRNA) is shown as a loading and quality control. Control represents phenazine-untreated mycelia. Full-length blots/gels are presented in the Supplementary Figure 4 (4A – RNA-gels and 4B – Northern blots; all the gels were run under the same experimental condition).

To further characterize the effects of 1-HP on the metabolism of iron in *A. fumigatus*, we studied the short-term impact of phenazines at the transcriptional level, by analyzing genes involved in iron responses. Northern blot analyses in figure 6 show that 1-HP caused an induction of genes previously shown to be induced by iron starvation including the iron regulator-encoding gene *HAPX*, the siderophore transporter-encoding gene *MIRB*, as well as the siderophore biosynthetic genes *SIDA*, *SIDF* and *SIDG*^27^,^28^. Inversely, 1-HP caused down-regulation of genes that have previously been shown to be repressed during iron-depleted conditions including aconitase-encoding *ACOA* and cytochrome *c*-encoding *CYCA*^27^,^28^ (Figure 6). These data clearly demonstrate that 1-HP causes an iron starvation response in *A. fumigatus*, which is in accordance with the 1-HP hypersensitivity of mutants impaired in adaptation to iron starvation as well as the previously shown 1-HP-mediated stimulation of siderophore production in *A. fumigatus*^17^. Taken together, these results indicated that these mechanisms were required to compensate the iron starvation caused by 1-HP and were in agreement with the increased susceptibility of mutants impaired in adaptation to iron starvation (Δ*hapX*, Δ*sidD* and Δ*sidF,* see above).

#### 1-HP is a chelator

**Figure 7:**
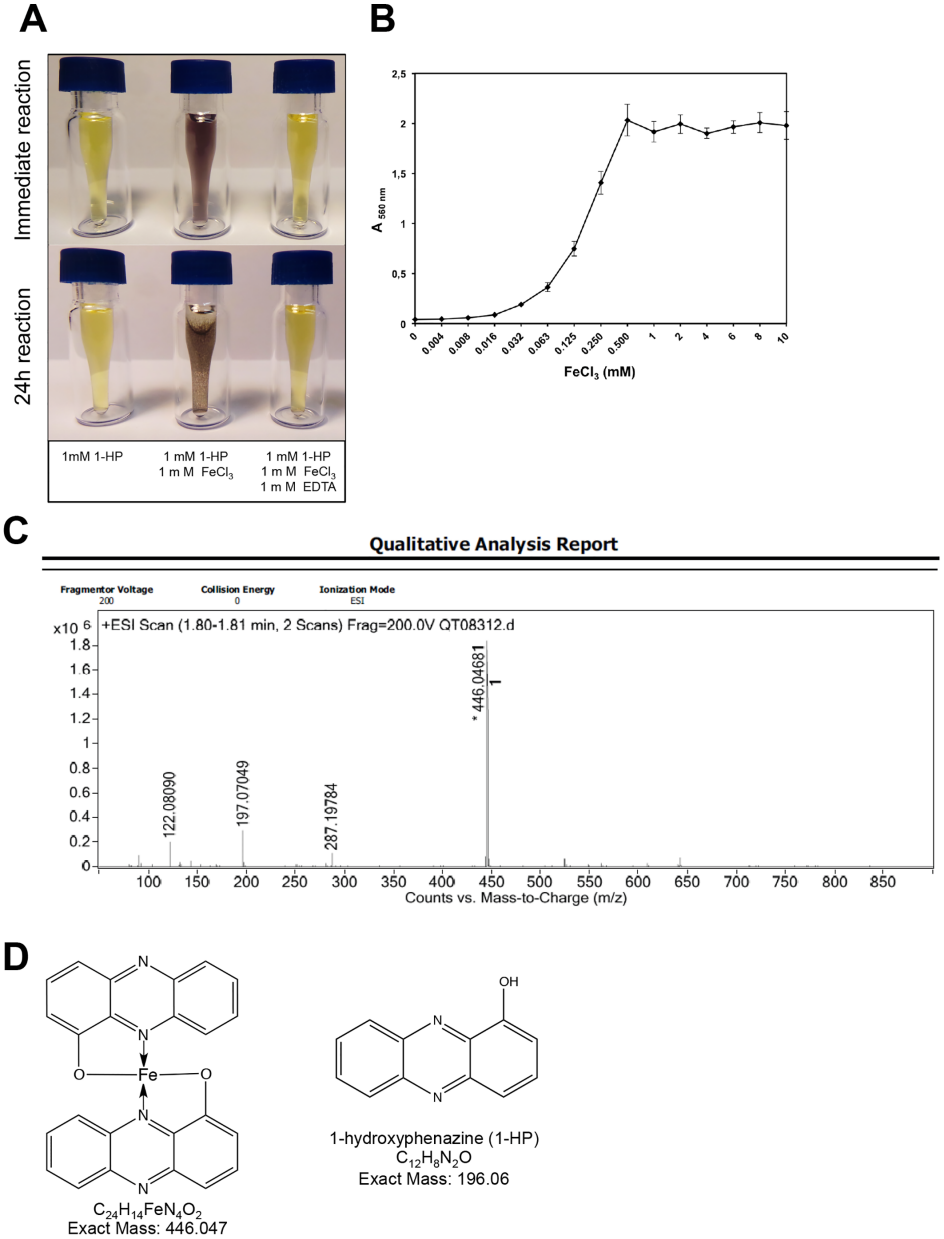
1-HP chelates iron. (A) Interaction of 1-HP with iron (FeCl_3_) in absence or presence of EDTA, showing an immediate change yellow colored 1-HP in the presence of iron and absence of EDTA, and flocculation of the [1-HP-iron] complex after 24 h incubation. (B) λ_560 nm _titration curve of the [1-HP-iron] complex in presence of increasing concentrations of FeCl_3_. (C) LC-MS analysis of the [1-HP-iron] flocculation product. (D) Deduced structures of the complexes formed by 1-HP and FeCl_3_ based on LC-MS analysis, compared to the exact mass and structure of 1-HP.

In the presence of FeCl_3_, we observed that the native yellow color of 1-HP turned immediately to purple, and the [1-HP−iron] complex flocculated in thirty minutes (Figure 7A). Addition of EDTA prevented the binding of iron to 1-HP, showing that EDTA has a higher affinity to iron than 1-HP (Figure 7A). On the other hand, none of the other phenazines chelated iron (data not shown).

The chelating activity of 1-HP was studied by titrating 1-HP against increasing concentrations of FeCl_3_ followed by appearance of the [1-HP−Fe] complex. This was determined by reading absorbance at 560 nm since it corresponded to maximal absorbance of the [1-HP−Fe] complex, whereas FeCl_3_ and 1-HP standalone did not absorb at this wavelength (data not shown). The absorbance of the complex prior to flocculation increased with the iron concentration (Figure 7B). In the presence of 1 mM 1-HP, we observed that the curve was exponential from 0 to 0.5 mM FeCl_3_ and the saturation point was obtained at 0.5 mM FeCl_3_ (Figure 7B). The flocculation product was recovered and analyzed by LC-MS. The main peak (m/z = 446.05) observed in ESI corresponds to a molecular ion where one iron ion is coordinated by two 1-HP and the mass corresponded to two molecules of 1-HP for one molecule of iron (Figure 7C). These results confirmed the stoichiometry and complex saturation concentration determined in the titration experiment (Figure 7B).

**Figure 8:**
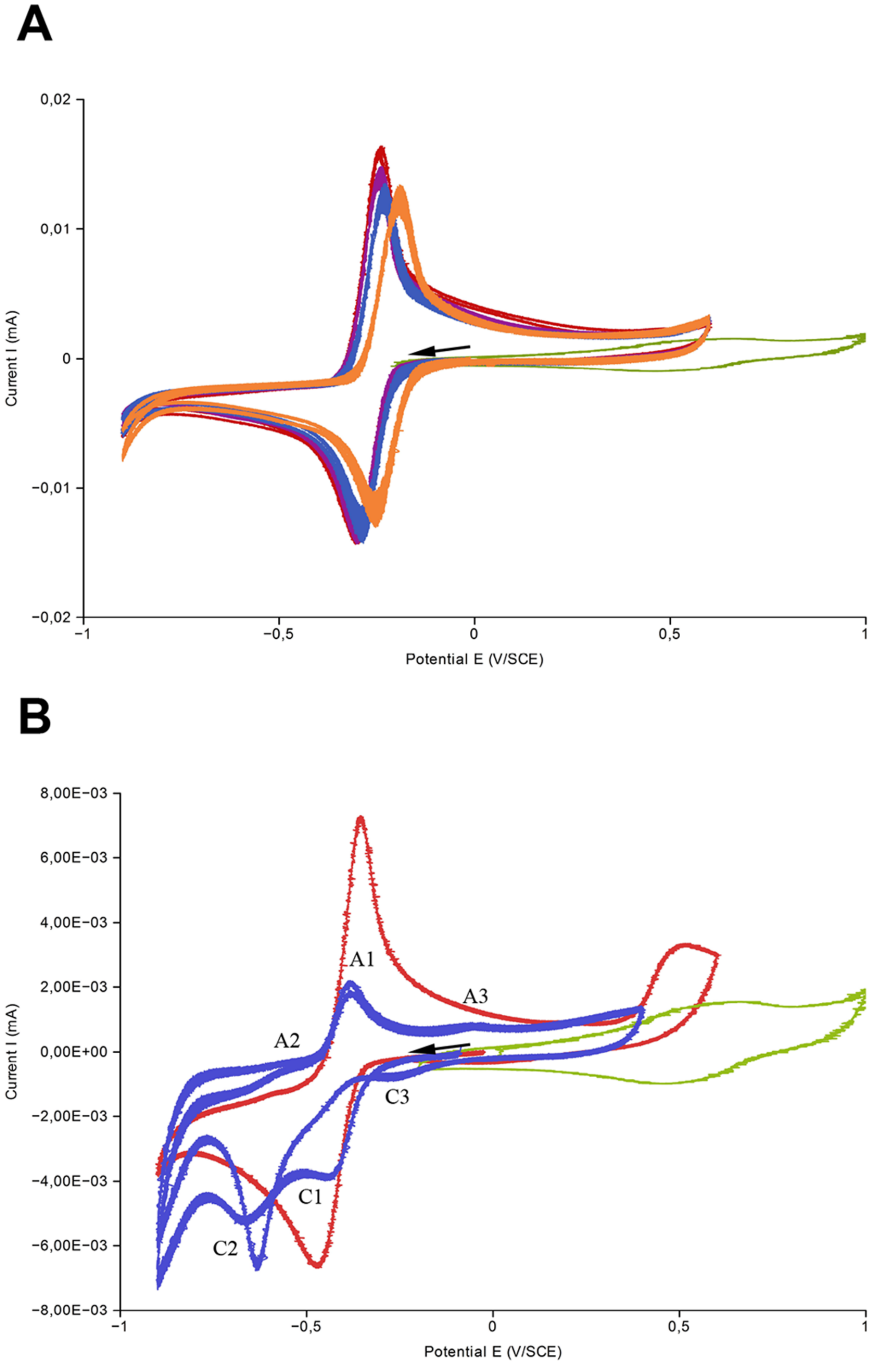
1-HP-iron complex is a quasi-irreversible redox system. (A) Cyclic voltammetry at 100 mV/s on a gold disc electrode with 1 mM of PYO (red), 1 mM of FeCl_3_ (green) and a mixture of PYO-FeCl_3_ in the ratio (1:0.1) in violet, (1:0.5) in blue and (1:1.5) in orange. (B) Cyclic voltammetry at 100 mV/s on gold disc electrode with 1 mM of 1-HP (red), 1 mM of FeCl_3_ (green) and a mixture of 1HP-FeCl_3_ (1:1) (blue). The arrow indicates the direction from initial potential.

The formation of the [1-HP−Fe] complex was further verified by cyclic voltammetry by successive additions of Fe(III) into 1-HP buffered solutions and compared with a non-iron-complexing phenazine, PYO. When Fe(III) was into the PYO solution, no significant change in the shape nor height of the peak observed. The reductive and the oxidative peaks were comparable regardless of Fe(III) concentration. The absence of any electrochemical effect following Fe(III) addition into PYO-buffered solutions suggests that PYO could not form any complex with iron (Figure 8A). In contrast, the cyclic voltammetry of 1-HP in the presence of Fe(III) is different (Figure 8B). Firstly, both the reductive and oxidative peaks were smaller and wider compared to peaks without Fe(III), with no significant difference in their location. This observation was visible only for equimolar additions of Fe(III). Two reductive peaks appeared (C1 and C2) and they were coupled with two oxidative peaks (A1 and A2). This cyclic voltammetry shape may be explained by a coupling of homogeneous chemical reactions with electrode electron transfers mechanism where the phenazine first reacts with iron before being reduced. Since a part of 1-HP had reacted with iron species, the reductive peak C1 corresponds to the free phenazine that was reduced. It was then oxidized at A1, and this system was quasi reversible. This system C1/A1 was lower than 1-HP without iron. The C2 peak could be the reduction of the [1-HP−Fe] complex. As the coupled A2 anodic peak was weak, this system appeared irreversible. On the return cycle, a third anodic peak (A3) appeared and was coupled with a reductive peak C3. Those last peaks probably corresponded to the Fe(III)/Fe(II) system which was shifted towards more reductive potentials due to the chemical chelation of iron species by 1-HP. With increasing iron concentrations C1 and A1 tended to become smaller whereas C2 became bigger – A2 stayed as a small peak (data not shown). However those electrochemical results did not give information on the oxidation state of the iron.

#### PYO, PCN and PCA promote acquisition of iron by A. fumigatus cells

**Figure 9:**
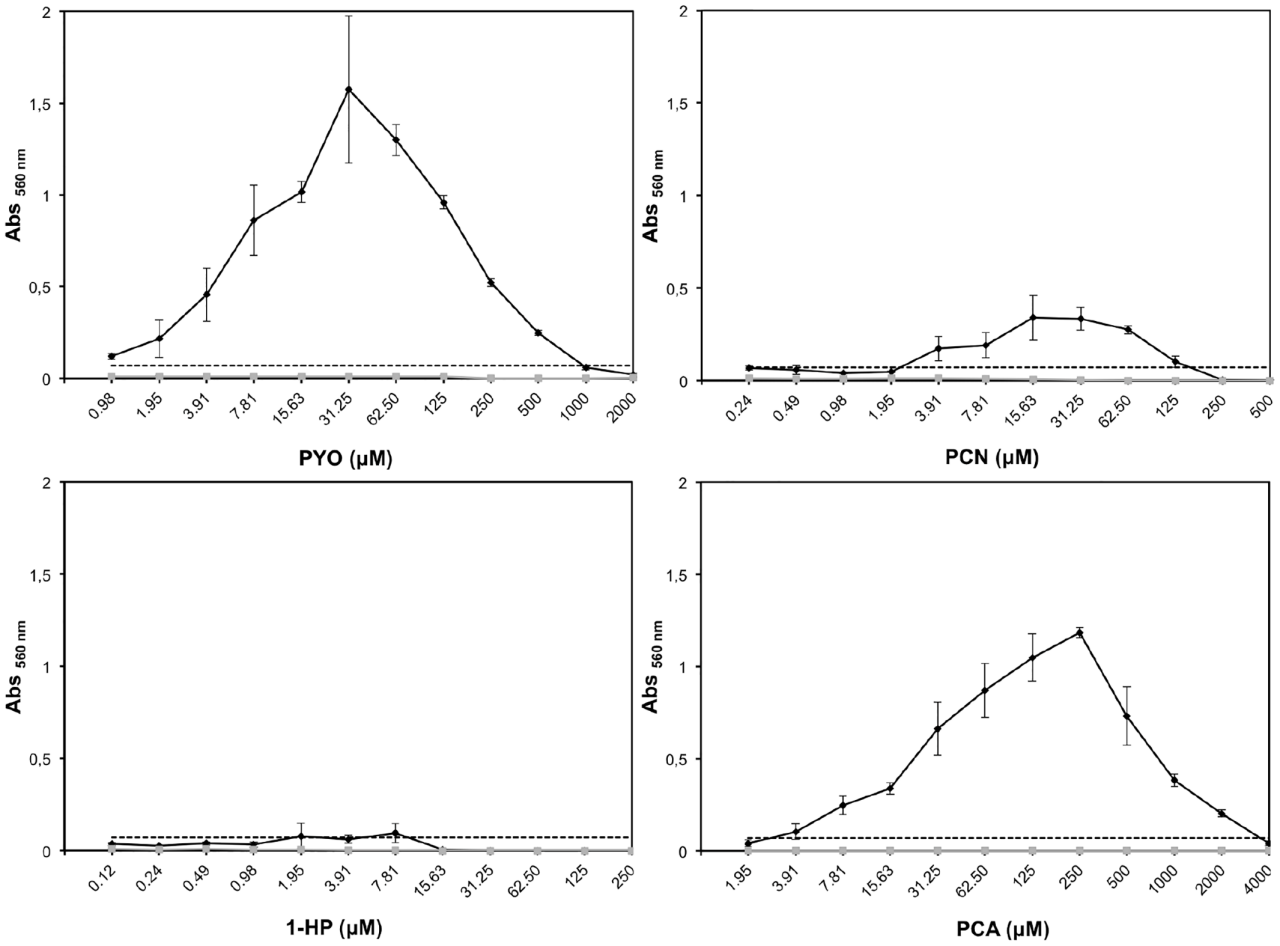
Growth of *A. fumigatus* Δ*sidA* is stimulated by low concentration of phenazines. (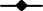) Δ*sidA* and (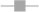) Δ*sidA*Δ*ftrA* mutants incubated in presence of increased concentration of phenazines. (– –) Δ*sidA* incubated in absence of phenazines. The growth was quantified at absorbance 560 nm following the crystal violet procedure.

Iron is also essential for *A. fumigatus* growth. The Δ*sidA* mutant, which is unable to produce siderophores showed a growth deficiency in media containing less than 10 μM FeSO_4_^25^. In 2YT medium, Δ*sidA* showed poor growth, suggesting that the accessible iron concentration was under 10 μM in this medium (Figure 9). However, in presence of sub-inhibitory concentrations of the phenazines PYO, PCN and PCA, the growth of Δ*sidA* was stimulated (Figure 9). This result suggested that at low concentrations, phenazines were able to improve iron acquisition by *A. fumigatus*, most likely by increasing iron solubility following reduction of Fe(III) to Fe(II). Figure 9 shows that Δ*sidA* was as susceptible as the parental strain to increased concentrations of PYO, PCN and PCA since complete growth inhibition was reached at the same MICs as the parental strain (Figure 9, Table 2). However, 1-HP did not stimulate Δ*sidA* growth, most likely due to its iron chelating ability. Accordingly, Δ*sidA*, which is the mutant displaying the highest susceptibility to iron starvation of all tested mutants, displays the lowest MIC of 1-HP (15.63 μM) (Figure 9, Table 2).

Fe(II) could penetrate in the *A. fumigatus* cells through the low-affinity ferrous iron uptake, which has not yet been identified at the molecular level yet, or Fe(II) is reoxidized and imported by the protein complex consisting of the ferroxidase FetCp and the ferric iron permease FtrAp^25^. In presence of sub-inhibitory concentrations of PYO, PCN and PCA, the growth defect of Δ*sidA*Δ*ftrA* in 2YT was not restored (Figure 9). This result shows that PYO, PCN and PCA generate Fe(II) by the reduction of Fe(III), which is then taken up by the FetC/FtrA complex and not by the low-affinity Fe(II) uptake system.

## Discussion

Phenazines are heterocyclic redox-active compounds that are produced naturally in a soluble reduced form. The aromatic ring is substituted by different functional groups, resulting in variously colored derivatives. The natural colors of the four phenazines of *P. aeruginosa* used in this study are yellow for PCN and PCA, orange for 1-HP and blue for PYO. The functional groups of phenazines also determine specific emission wavelength absorbance, redox potential and solubility of the molecule, thus affecting the biological activity^1^. The antagonistic effects of almost all phenazines are usually attributed to one general characteristic: redox activity^1^. In our study, we chose to focus on PYO, PCN, 1-HP and PCA because they are the most important phenazines of *P. aeruginosa* in its interactions with *A. fumigatus*^17^.

Previous studies characterized the electrochemical properties of the four phenazines, showing that the half-wave potentials (E_1/2_) values of these phenazines gave rise to a classification established from the least to most reductive phenazine^35^. PYO E_1/2_ was the lowest followed by those of PCA, PCN and 1-HP^35^. Except for PCA, this classification mirrors that of the MICs of the phenazines, with our data showing that the MIC of PYO, PCN and 1-HP against *A. fumigatus* were correlated to their redox potentials. PCA presented a MIC lower than PYO but we demonstrated that PCA was not very active at pH 7 due to its negative charge. In pH 5, wherein the PCA is not negatively charged but neutral, the MIC was greatly reduced, compared to the MIC obtained at pH 7, which confirms the lower ability of this phenazine to penetrate the cell under the latter condition.

Reduced phenazines are oxidized in the target cell by oxygen and NAD(P)H, generating ROS. The many effects of PYO and PCA on diverse eukaryotic hosts and prokaryotes are thought to result from their redox activities or inactivation of proteins important in the oxidative stress response^7^,^8^,^36^. Similar results were found for phenazines secreted by other *Pseudomonas* sp, e.g., the 2-hydroxyphenazine-1-carboxylic acid (2-OHPCA) produced by *P. aureofaciens* is thought to kill off competing fungi through the production of ROS^37^. Using the oxidant-sensitive probe H_2_DCFDA, we demonstrated for the first time that the four major phenazines of *P. aeruginosa* produced ROS following penetration of *A. fumigatus* swollen conidia and hyphae (Figure 4). We showed that phenazines had a dramatic impact on the ultrastructure of mitochondria of *A. fumigatus* hyphae and that Sod2p, which is the mitochondrial superoxide dismutase, is essential for the resistance to phenazines. Moreover, mitotracker that stains only cells harboring active mitochondria did not label phenazines-treated *A. fumigatus* cells. These data suggested that the main ROS target of phenazines is the mitochondria. Our results are supported by previous data which reported that PYO decreased mitochondrial membrane potential, aconitase activity and ATP levels, and altered the ultrastructure of mitochondria in epithelial lung cells^8^. Similar to *A. fumigatus* hyphae, mitochondrial MnSod2p was involved in the resistance of epithelial lung cell to PYO as demonstrated by the decrease in the PYO-induced damages after overexpression of this superoxide dismutase^8^. Mutant strain analysis showed that cytoplasmic Sodp (Sod1p and Sod3p) play a minor role in the resistance of *A. fumigatus* to phenazines since the triple Δ*sod1*Δ*sod3*Δ*sod2* mutant, but not the Δ*sod1* or Δ*sod3* single mutants, displayed higher susceptibility than Δ*sod2*. Mitochondrial alteration leads to the release of ROS into the cytoplasm, as observed by the diffuse cytoplasmic DCF-fluorescence, which could explain why the absence of all superoxide dismutases in the triple Δ*sod1*Δ*sod3*Δ*sod2* mutant led to a hypersensitivity to phenazines. Superoxide dismutases act by converting O_2_^**·**−^ in H_2_O_2_ which is further detoxified by the H_2_O_2_ scavenging system, glutathione, thioredoxine, and catalases regulated by the two transcription factors Yap1p and Skn7p. Mutants deleted in catalases, *YAP1* and *SKN7* were not any more sensitive to phenazines than the parental strain (Table 1), showing that the scavenging of H_2_O_2_ was catalase-independent after phenazine stress in *A. fumigatus*.

Using the reactive nitrogen species sensitive dye DHR123, we also demonstrated that phenazines induce RNS in *A. fumigatus* cells. RNS are associated with pathogen killing in macrophages^38^. The production of ROS and RNS are intimately linked, the first step being the production of nitric oxide (NO^**·**^) from the reduction of nitroxyl anion (NO^−^). In presence of increased concentration of O_2_^**·**−^, such as in presence of phenazines, NO^**·**^ reacts with O_2_^**·**−^ to form peroxynitrite (ONOO^−^)^39^,^40^. ONOO^−^ itself is a highly reactive species which can directly react with various biological targets and components of the cell including proteins such mitochondrial MnSodp, lipids, thiols, amino acid residues, DNA bases, and low-molecular weight antioxidants^32^.

Compared to other phenazines, the PCA-treated *A. fumigatus* cells showed most altered mitochondria (Figure 2) and highest DCF- and Rhodamine 123-fluorescent spots (Figures 4,5). These data and the mitotracker staining suggested that PCA induced a major production of ROS and RNS in mitochondria. In addition, the deletion of mitochondrial superoxide dismutase (*SOD2*) was more detrimental as Δ*sod2* mutant was at least eight times more sensitive to PCA than the WT (Table 1).

1-HP was found to be the most active phenazine against *A. fumigatus*. In addition to the ROS production, its high inhibitory activity is due to a characteristic specific to this phenazine. We demonstrate for the first time that 1-HP is able to chelate iron. Previous data showed that 1-HP induces the production of the extracellular siderophores FsC and TAFC by *A. fumigatus*^17^. Here we found that (i) mutants impaired in iron adaptation (Δ*hapX* Δ*sidD* and Δ*sidF*) are hypersusceptible to 1-HP, (ii) addition of TAFC reduces the inhibitory activity of 1-HP against Δ*hapX*, Δ*sidD* and Δ*sidF*, (iii) 1-HP transcriptionally induces an iron starvation response, i.e. it induces genes required for adaptation to iron starvation (*HAPX, SIDA, SIDF, SIDG*, *MIRB*) and represses genes encoding iron-dependent proteins (*ACOA, CYCA*) and (iv) 1-HP is able to chelate iron (Figure 8). Taken together, these results strongly suggest that 1-HP causes iron starvation in *A. fumigatus* by chelating iron, which is partially compensated by the fungal enzymatic and regulatory mechanisms involved in adaptation to iron starvation. As shown before, PYO, PCA and PCN were not found to be chelators^41^.

One idea that has emerged in the recent years is that PYO and PCA can beneficially modulate bacterial biofilm development and iron acquisition^13^,^41^,^42^. *In vivo*, different host innate immunity proteins such as transferrin or lactoferrin sequester Fe(III), which potentially blocks *P. aeruginosa* biofilm formation or *A. fumigatus* growth at early stages of infection^13^. *In vitro* experiments have shown that *Pseudomonas* phenazines can liberate Fe(III) from transferrin by reducing it to ferrous iron Fe(II), which is taken up by the bacterial cells via the ferrous iron transporter^13^,^41^. Like *P. aeruginosa*, *A. fumigatus* employs three iron uptake mechanisms: (i) low-affinity ferrous iron uptake, (ii) high-affinity reductive iron uptake starting with reduction of Fe(III) to Fe(II) by metalloreductases followed by its uptake by a protein influx complex, and (iii) siderophore-mediated iron uptake^25^. Using the *A. fumigatus* mutant lacking SidAp, which is required for extra- and intracellular-siderophore biosynthesis, we demonstrated that sub-inhibitory concentrations of the three phenazines PYO, PCA and PCN induced *A. fumigatus* growth in iron starvation conditions. Most likely, PYO, PCA and PCN increase, via their redox activity, the solubility of iron by reduction of Fe(III) to Fe(II), which is taken up by the FetCp/FtrA complex.

Iron is an important environmental parameter that helps pathogens thrive in sites of infection. In cystic fibrosis patients infected by *P. aeruginosa*, phenazines may facilitate Fe(III) reduction *in vivo*, as evidenced by the generally high percentage of Fe(II) once phenazine levels rise above ~50 μM in expectorated sputum^43^. The maintenance of a bioavailable Fe(II) pool facilitates *P. aeruginosa* biofilm formation^13^. *A. fumigatus* infection is found in many cystic fibrosis patients following *P. aeruginosa* infection^14^. One explanation could be that *P. aeruginosa* facilitates *A. fumigatus* growth in these patients. PYO and PCA have been found *in vivo* at concentrations in the range of 1 to 100 μM, which we demonstrated to be subinhibitory against *A. fumigatus*. Moreover, these PYO, PCA and PCN concentrations were optimal to stimulate iron-acquisition and consequently, growth of *A. fumigatus*. In addition, iron chelation by 1-HP will stimulate the TAFC secretion by *A. fumigatus* and consequently fungal growth.

**Figure 10:**
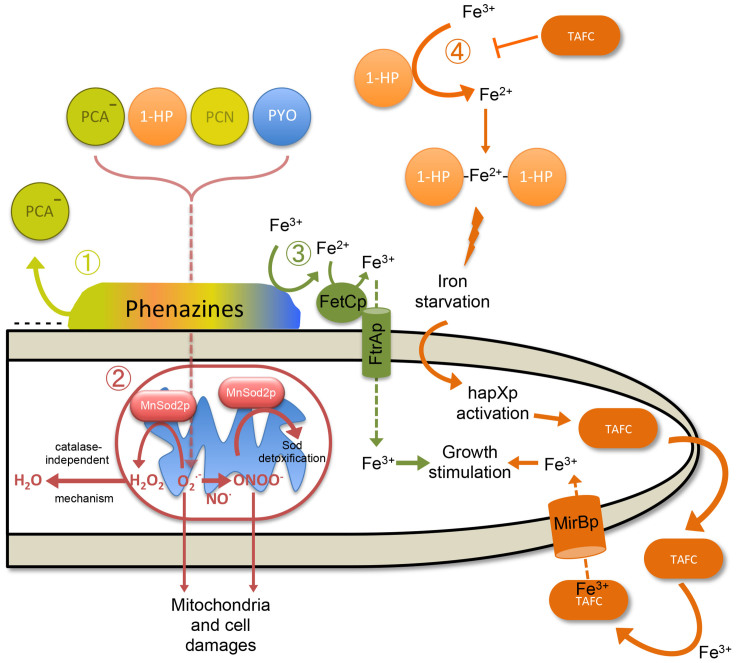
Model for phenazines mode of action against *A. fumigatus*. The four major phenazines of *P. aeruginosa* bind to hyphae and penetrate the cell. Few PCA penetrates due its negative charge (Lime green pathway,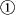). Phenazines act at the level of mitochondria and induce the production of superoxide anion (O_2_^**·**−^) and peroxynitrite (ONOO^−^) (Red pathway,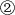). MnSod2p enzyme converts O_2_^**·**−^ to hydrogen peroxide (H_2_O_2_) and a catalase-independent mechanism allows its detoxification into H_2_O. The anion ONOO^−^ formed from O_2_^**·**−^ and nitric oxide (NO^**·**^) is also detoxified by MnSod2p (Red pathway,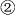). PYO, PCN and PCA reduce Fe(III) to Fe(II) which penetrates the *A. fumigatus* cell through the iron ferroxidase FetCp/permease FtrAp complex (Green pathway,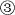). 1-HP also reduces Fe(III) to Fe(II) and two 1-HP molecules can chelate the newly formed Fe(II) (Orange pathway,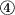). This chelating activity induces iron starvation which causes HapXp activation. The biosynthetic pathway of triacetylfusarinine C (TAFC) is then activated and allows Fe^3+^ acquisition to stimulate the growth of the fungus (Orange pathway,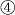).

An important highlight of this work shows at low concentrations, the phenazines PYO, PCA and PCN were stimulating the growth of *A. fumigatus* because they were reducing the Fe(III) and were promoting the fungal growth in iron-starved environment (green pathway Figure 10). At high concentration, the four phenazines have anti-*A. fumigatus* activities because they are able to penetrate into the cells and induce the production of ROS and RNS mainly in the mitochondria leading to mitochondrial alterations and fungal death (red pathway Figure 10). *A. fumigatus* superoxide dismutase Sod2p is essential for the detoxification of these reactive species. The fourth phenazine 1-HP had a mode of action completely different from PYO, PCA and PCN: 1-HP is an iron chelator that represses fungal growth in low iron environment (orange pathway Figure 10). Our data show for the first time the fine tuning in the interactions existing between *A. fumigatus* and the different concentrations and chemical composition of the phenazines of *P. aeruginosa* which can lead to stimulatory or antagonistic biological effects.

## Methods

### Strains and culture conditions

*A. fumigatus* strains used in this study are listed in Supplementary Table 2. The *A. fumigatus* reference strain used in this study was CEA17Δ*akuB*^KU80^ mutant deficient in non-homologous end joining^44^. It originates from a clinical strain and is pathogenic in experimental murine aspergillosis models. All strains except mutants in siderophore pathways, were conserved on 2% (w/v) malt agar slants. Mutants in the siderophore pathways were conserved on *Aspergillus* Minimal Media (+Fe-AMM; iron abundant conditions) according to Pontecorvo *et al.*^45^ containing 1% (wt/vol) glucose as carbon source, 20 mM glutamine as nitrogen source and 0.03 mM FeSO_4_. 1 week-old conidia were recovered from the slants by vortexing with 0.05% (v/v) Tween 20 aqueous solution and used for inoculation of 2YT medium containing 16 g L^−1^ Bactotryptone, 10 g L^−1^ Bacto Yeast Extract, 5 g L^−1^ NaCl, 2 g L^−1^ Glucose at pH 7 at 37°C.

### Chemicals products

Commercial *P. aeruginosa* phenazines were used in this study. PYO was purchased from Sigma Aldrich, PCN from Princeton Biomolecular Research, 1-HP from TCI Europe N.V. and PCA from Apollo Scientific. PYO and 1-HP were diluted in methanol. PCN and PCA were diluted in DMSO.

### Phenazine susceptibility assays

The susceptibility of *A. fumigatus* to phenazines was undertaken by measuring the MIC of each molecule in 96-well flat bottom plates, using a modified protocol of Clavaud *et al*.^46^. Briefly, the assay mixture was prepared by adding 1 volume of conidial suspension (1 × 10^5^ conidia ml^−1^ in 0.05% Tween 20-water) to 12 volumes of assay medium (2YT containing 0.1% Tween 20, and 1% methanol or 1% dimethyl sulfoxide (DMSO) depending on the phenazine used). Two-fold dilutions of phenazines were prepared with this assay mixture. The plates were incubated at 37°C for 20 h. Biofilm biomass was assessed using a modified protocol of O'Toole^47^. After incubation, the culture medium was removed from each well and the biofilms were washed three times with Milli-Q water. 130 μl of 0.01% (w/v) crystal violet solution (Sigma Aldrich) was added to each well for 20 min at room temperature. The solution was then removed by carefully rinsing the biofilms under running Milli-Q water until the supernatant was clear. The plates were air-dried overnight. The biofilms were destained by the addition of 130 μl of 30% acetic acid in water to each well for 20 min under agitation at 250 rpm. The acetic acid was removed to clean 96 well plates and the absorbance at 560 nm was measured (Thermo Labsystems Multiskan EX).

### Microscopic analyses of cell morphology

The effect of phenazines on the morphology of *A. fumigatus* hyphae grown as described above (*Phenazine susceptibility assays*) was visualized by light microscopy.

For Transmission Electron Microscopy, *A. fumigatus* was grown on 8 well 15 μm-slides (ibidi) in presence of 1 mM of PYO, 0.125 mM of PCN, 62.5 μM of 1-HP or 2 mM of PCA (MIC_50_ concentrations) during 18 h at 37°C. Hyphae were fixed with 2.5% glutaraldehyde in 0.1 M Cacodylate buffer pH 7.2 overnight at 4°C. Specimens were postfixed successively with 1% tannic acid and a mix of 1% osmium tetroxide and 1.5% potassium ferrocyanide for 1 h at room temperature, dehydrated in ethanol gradient, and embedded in Epon. Thin sections were cut with a Leica Ultramicrotome Reichert Ultracut S, stained with uranyl acetate and lead citrate. Images were taken with a Tecnai Electron Microscope at 100 kV.

### Phenazine staining of mycelia

1 × 10^7^ conidia ml^−1^ were inoculated into 2YT medium for 14 h at 37°C under shaking conditions (150 rpm). 1 ml of this culture was transferred into 24-well plates (TPP) and incubated with 1 mM of each phenazine for 6 h at 37°C under shaking conditions. The mycelium was recovered by filtration, washed three times with Milli-Q water and observed under light microscopy.

### Fluorescence microscopy of phenazine penetration

To visualize the penetration of phenazines in *A. fumigatus* cells, swollen conidia obtained as below (**ROS and RNS assays**) were incubated with 2 mM PYO, 0.25 mM PCN, 0.125 mM 1-HP or 4 mM PCA (MIC concentrations) in 2YT medium for 1 h at 37°C. The natural fluorescence of the phenazines under their reduced state was observed with an inverted fluorescence microscope Zeiss Apotom Observer Z1, at a filter excitation BP 365 nm, FT 395 nm and emission LP 397 nm.

### ROS and RNS assays

To detect ROS and RNS in fungal structures, 1 × 10^7^ conidia per ml were incubated into 2YT medium pH 7 at 37°C under shaking for 4 h 30 min. 2.5 μg ml^−1^ of the O_2_^**·**−^ fluorescent probe H_2_DCFDA or 10 μM of the peroxynitrite fluorescent probe DHR123 were added to the swollen conidia for 30 min in the dark at 37°C, followed by the addition of each phenazine at the MIC concentration. The incubation was continued for 1 h in the dark at 37°C. After washing, fluorescence of the respective reduced products DCF and Fluorescein 123 was visualized using a fluorescence microscope Leica DMLB with Leica filter I3, a filter excitation BP 450–490 nm, FT 510 nm and emission LP 515 nm.

### Analysis of the 1-HP-Fe complex

The addition of FeCl_3_ in presence of 1-HP modified the color of solution from yellow to dark violet, characteristic of the formation of a complex. The specificity of the chromophore formed was scanned by spectrophotometry and maximal absorption was obtained at 560 nm. The titration was done using 1 mM 1-HP in 5 mM Tris-HCl pH 7 containing 50 mM NaCl (Tris-Nacl buffer) and 0 to 10 mM FeCl_3_.

To analyze the 1-HP-metal ion complexes, 1 mM 1-HP was incubated in presence of 1 mM of FeCl_3_ in Tris-NaCl buffer. The reaction was continued until flocculation of the complexes was completed. After 24 h, the flocculated products were recovered and freeze-dried. The flocculated products were solubilized in chloroform and analyzed by LC-MS in the Agilent 1200 with analyzer tandem Q-Tof, Agilent Accurate Mass QToF 6520.

### Voltammetry experiments

The voltammetry experiments were performed as previously described by Wang and Newman^35^, with the following modifications. Electrochemical studies were performed using a classical three-electrode cell with a Saturated Calomel Electrode (SCE) as a reference electrode (*E*(SCE) = 0.244 V/SHE), a platinum wire as counter electrode and a disk-shaped gold electrode (3 mm diameter) as the working electrode. Working electrodes were polished before each experiment with 2400 and 4000 grit SiC papers and then thoroughly rinsed with water. All the experiments were carried out under argon bubbling to avoid dissolved oxygen, at room temperature. Electrochemical data were collected using a BioLogic Science Instruments VSP Potentiostat with EC-Lab software. All the electrochemical data were determined from cyclic voltammetry (CV) results.

1 mM of PYO or 0.5 mM of 1-HP was solubilized in 10 mM MOPS buffer pH 7, containing 10 mM ammonium acetate and 0.1 M KCl. The FeCl_3_ solution was the source of Fe(III) ions. The concentration of iron added corresponded to stoichiometric ratios with phenazine as follows: 1:0.1; 1:0.5; 1:1; 1:1.5.

### Northern Blot Analysis

*A. fumigatus* was grown for 12 h in liquid 2YT medium, followed by the addition of 0.0625 mM 1-HP and incubated for an additional 2 h. Mycelia were harvested, total RNA was isolated using TRI Reagent (Sigma) and 10 μg were used for the analysis as described previously^48^. Hybridization probes were generated by PCR amplification-labeling with Digoxigenin (Roche) using the primers listed in Supplementary Table 3. As 1-HP is solubilized in methanol, this substance was added to the cultures as a control.

## Supplementary Material

Supplementary Information Supplemental data revised

## Figures and Tables

**Table 1:** Phenazine Minimal Inhibitory Concentration (MIC) of oxidative stress mutants. Response of H_2_O_2_ stress-mutants (Δ*yap1*, Δ*skn7*, catalase-deleted mutants (Δ*cat*)) and O_2_^**·**−^ scavenger-mutants (superoxide dismutases; Δ*sod*)

		Phenazines MIC (mM)			
Strain name	Parental strain	Pyocyanin (PYO)	Phenazine-1-carboxamide (PCN)	1-Hydroxyphenazine (1-HP)	Phenazine-1-carboxylic acid (PCA)
Parental strain 1: CEA17 Δ*akuB*^KU80^	CBS 144.89	2	0.25–0.5	0.125–0.25	≧4
Δ*yap-1*	CEA17 Δ*akuB*^KU80^	"	"	"	"
Δ*skn7*	CBS 144.89	"	"	"	"
Parental strain 2: G10	CBS 144.89	"	"	"	"
Δ*cat1*	G10	"	"	"	"
Δ*cat2*	G10	"	"	"	"
Δ*catA*	G10	"	"	"	"
Δ*cat1*Δ*cat2*	G10	"	"	"	"
Δ*cat1*Δ*catA*	G10	"	"	"	"
Δ*sod1*	CEA17 Δ*akuB*^KU80^	"	"	"	"
Δ*sod2*	CEA17 Δ*akuB*^KU80^	0.25–0.5	0.0625–0.125	0.0313–0.0625	0.5–1
Δ*sod3*	CEA17 Δ*akuB*^KU80^	2	0.25–0.5	0.125–0.25	≧4
Δ*sod1*Δ*sod3*	CEA17 Δ*akuB*^KU80^	"	"	0.0625–0.125	"
Δ*sod1*Δ*sod2*Δ*sod3*	CEA17 Δ*akuB*^KU80^	0.125–0.25	0.3125–0.625	"	0.5–1

**Table 2:** Phenazine Minimal Inhibitory Concentration (MIC) of siderophore mutants (Δ*sid*) and mutants in iron-stress responses (Δ*sreA*, Δ*hapX*). Grey boxes indicate addition of TAFC to final concentration of 10 μM

strain name	parental strain	Phenazines MIC (mM)			
		Pyocyanin (PYO)	Phenazine-1-carboxamide (PCN)	1-Hydroxyphenazine (1-HP)	Phenazine-1-carboxylic acid (PCA)
ATCC 46645	CBS 144.89	2	0.25–0.5	0.125–0.25	≧4
Δ*hapX*	ATCC 46645	"	"	0.0625–0.125	"
Δ*sreA*	ATCC 46645	"	"	0.125–0.25	"
Δ*sidA*	ATCC 46645	"	"	0.0156–0.0313	"
Δ*sidC*	ATCC 46645	"	"	0.125–0.25	"
Δ*sidD*	ATCC 46645	"	"	0.0313–0.0625	"
Δ*sidF*	ATCC 46645	"	"	"	"
ATCC 46645	CBS 144.89			0.125–0.25	
Δ*sidD*	ATCC 46645			"	
Δ*sidF*	ATCC 46645			"	
