# Supplementary material for: Pseudomonas aeruginosa manipulates redox and iron homeostasis of its microbiota partner Aspergillus fumigatus via phenazines
Source: Sci Rep. 2015 Feb 10;5:8220. doi: 10.1038/srep08220 (PMC5389140; doi:10.1038/srep08220)
Supplement: Supplementary Information — Supplemental data revised [file srep08220-s1.pdf]

Supplementary data

Pseudomonas aeruginosa manipulates redox and iron homeostasis of its microbiota partner *Aspergillus fumigatus* via phenazines

Benoit Briard[1,2], Perrine Bomme[3], Beatrix E. Lechner[4], Gaëtan L. A. Mislin[5], Virginie Lair[6], Marie-Christine Prévost[3], Jean-Paul Latgé[1], Hubertus Haas[4], Anne Beauvais[1,*]

Supplementary Figure 1

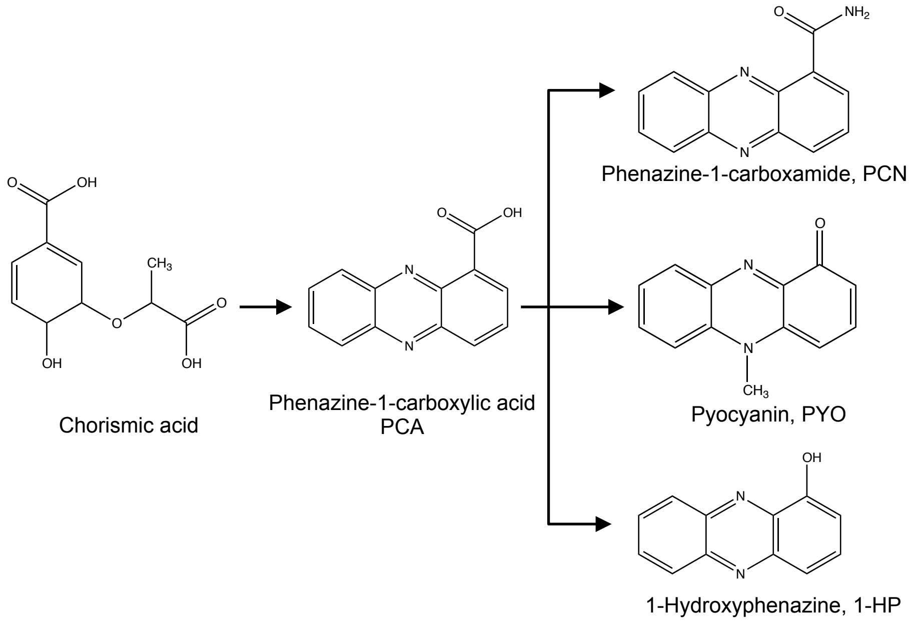

Supplementary Figure 1. **Biosynthesis of *P. aeruginosa* phenazines**

Supplementary Figure 2

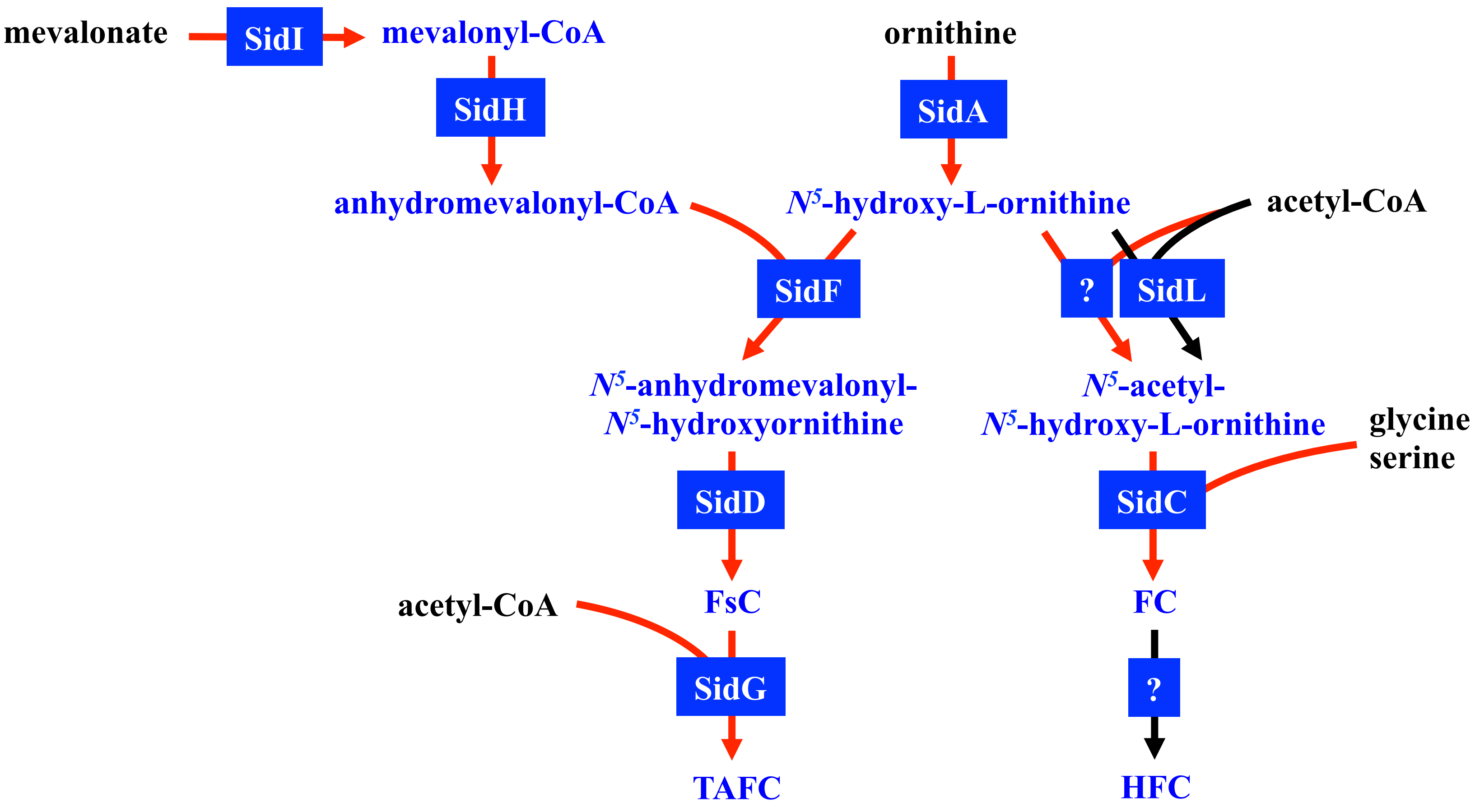

Supplementary Figure 2. A. fumigatus **siderophore biosynthetic pathway**
Siderophore pathway specific enzymes are boxed in blue. Enzymatic steps transcriptionally upregulated during iron starvation are marked by red arrows.

Supplementary Figure 3

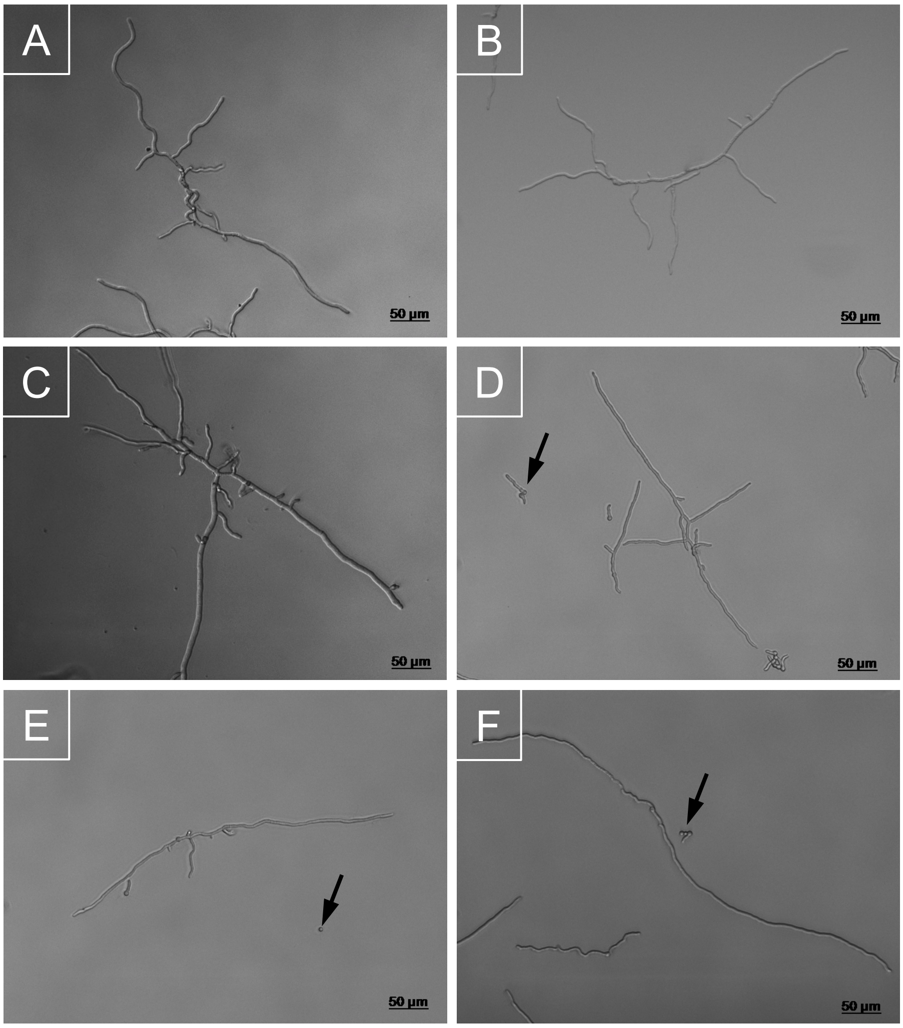

Supplementary Figure 3. **Morphology of *A. fumigatus* hyphae growing in presence of phenazines**

(A, B) control hyphae after 20 h growth at 30°C in presence of 1% DMSO (A) or 1% methanol (B). (C- F**)** *A. fumigatus* conidia incubated for 18 h at 37°C in presence of 2 mM PCA (C), 1 mM PYO (D), 62.5 µM 1-HP (E) or 125 µM PCN (F). Black arrows show swollen conidia or germ tubes indicating the heterogeneous growth of *A. fumigatus* in presence of phenazines.

Supplementary Figure 4

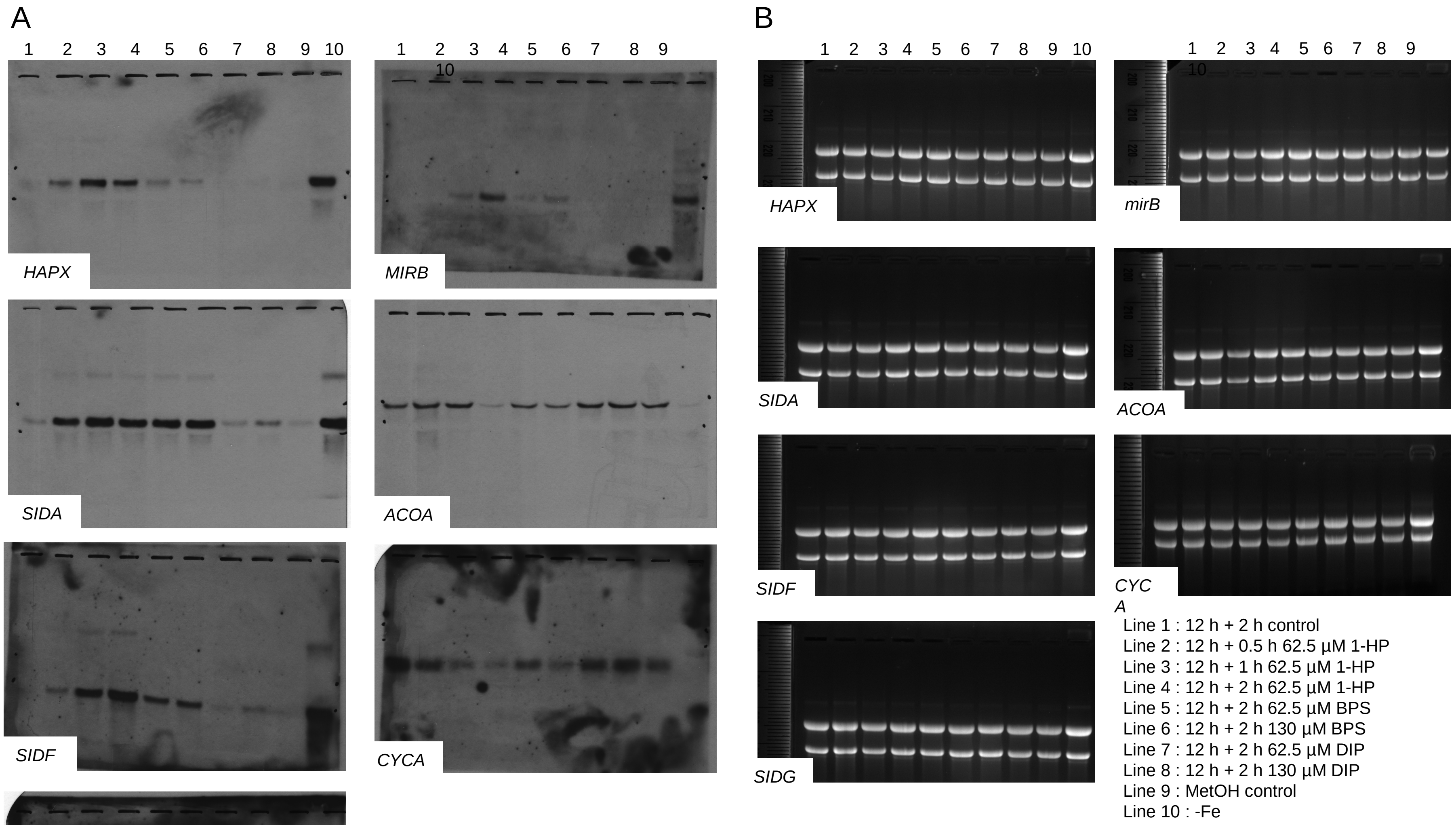

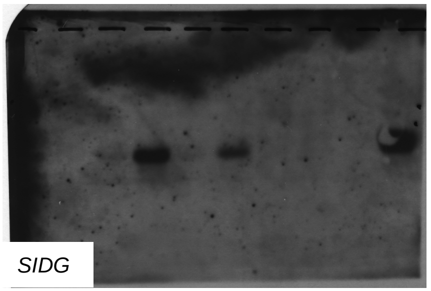

Supplementary Figure 4.* 1-HP induces an iron starvation response in *A. fumigatus. (A) Uncropped RNA-gels representing the quality and quantity of RNA used and (B) Uncropped Northern blots representing the expression of each gene. Two internal control were included, BPS (bathophenanthroline dissulfonate) and DIP (dipyridyl) which are respectively membrane impermeable and permeable ferrous iron chelators. The results showed that the induction of iron starvation at 2 hours of incubation was stronger with 1-HP compared to BPS, in contrast, DIP did not cause strong iron starvation in *A. fumigatus*.

Supplementary Table 1. pH dependent inhibitory effect of phenazines on the *A. fumigatus* CEA17Δ*akuB*^{KU80} grown in a set of 2YT medium buffered between pH 5 and pH 7

**Phenazine (abbreviation)**	**MIC (mM) pH5**	**MIC (mM) pH6**	**MIC (mM) pH7**
Pyocyanin (PYO)	2	2	2
Phenazine-1-carboxamide (PCN)	0.125-0.25	0.25-0.5	0.25-0.5
1-Hydroxyphenazine (1-HP)	0.0313-0.0625	0.0625-0.125	0.125-0.25
Phenazine-1-carboxylic acid (PCA)	0.25-0.5	2-4	≧4

Supplementary Table 2. Fungal strains used in this study

**Strain**	**Genotype**	**Reference**
Parental strain 1: CEA17Δ*akuB*^{KU80}	CEA17Δ*akuB*^{KU80}	[1]
Δ*sod1*	CEA17Δ*akuB*^{KU80}Δ*sod1::hph*	[2]
Δ*sod2*	CEA17Δ*akuB*^{KU80}Δ*sod2::hph*	[2]
Δ*sod3*	CEA17Δ*akuB*^{KU80}Δ*sod3::hph*	[2]
Δ*sod1*/Δ*sod3*	CEA17Δ*akuB*^{KU80}Δ*sod1::ble; Δsod3::hph*	[2]
Δ*sod1*/Δ*sod2*/Δ*sod3*	CEA17Δ*akuB*^{KU80}Δ*sod1::ble; Δsod2:ptrA; Δsod3::hph*	[2]
Parental strain 2: G10	Wild-type	[3]
Δ*catA*	G10Δ*catA::phleoR*	[4]
Δ*cat1*	G10Δ*cat1::hph*	[5]
Δ*cat2*	G10Δ*cat2::phleoR*	[4]
Δ*cat1*/Δ*cat2*	G10Δ*cat1::hph*;Δ*cat2::phleoR*	[4]
Δ*catA*/Δ*cat1*	G10Δ*catA::phleoR*;Δ*cat1::hph*	[4]
Parental strain 3: CBS 144-89	Wild-type	The Centraalbureau voor Schimmelcultures
Δ*skn7*	CBS 144-89Δ*skn7::hph*	[6]
Parental strain 4: ATCC 46645	Wild-type	American Type Culture Collection
Δ*yap1*	CEA17Δ *akuB*^{KU80}Δ*yap1::hph*	[7]
Δ*hapX*	ATCC 46645Δ*hapX::hph*	[8]
Δ*sreA*	ATCC 46645Δ*sreA::hph*	[9]
Δ*sidA*	ATCC 46645Δ*sidA::hph*	[10]
Δ*sidC*	ATCC 46645Δ*sidC::hph*	[10]
Δ*sidD*	ATCC 46645Δ*sidD::hph*	[10]
Δ*sidF*	ATCC 46645Δ*sidF::hph*	[10]
Δ*sidA*/Δ*ftrA*	CEA17Δ*sidA::pyrG; ΔftrA::hph*	[11]

Supplementary Table 3. Primers for amplification of the hybridization probes used in this study

**Accession number**	**Sequence 5' – 3'**	**Application**
AFUA_6G12930	CAG CGT CCT CTC ACA TAC	*ACOA*
	GCA AGA ACC GAT CAG ACC	
AFUA_0G13110	CCC TTT CTT GCA GTG TCC	*CYCA*
	CCG CGC ATC TGC TTT TAC	
AFUA_5G03920	TCG GTG GAA AGA AGT GCC	*HAPX*
	CGA CGA TGT ATT GTT ATT GG	
AFUA_3G03640	AAG CCG AGA AAA AGG GGG	*MIRB*
	AAC CCA GAT GAA GCC CAG	
AFUA_2G07680	AAC TAC CTC CAC CAG AAG	*SIDA*
	GAA CGG CAA TGT TGT AAG	
AFUA_3G03400	CCT CAT CCC TAT CTC ACC	*SIDF*
	AGT TTT GAG CGA GAG GGG	
AFUA_3G03650	ACA ATC AAG GCT CAG CCC	*SIDG*
	ACT TCG AGT CAT GCT GGG	

Supplementary References:

1. Ferreira, M. E. da S. *et al.* The akuBKU80 Mutant Deficient for Nonhomologous End Joining Is a Powerful Tool for Analyzing Pathogenicity in Aspergillus fumigatus. *Eukaryot. Cell* **5,** 207–211 (2006).
2. Lambou, K., Lamarre, C., Beau, R., Dufour, N. & Latge, J.-P. Functional analysis of the superoxide dismutase family in Aspergillus fumigatus. *Mol. Microbiol.* **75,** 910–923 (2010).
3. Monod, M. *et al.* Virulence of alkaline protease-deficient mutants of Aspergillus fumigatus. *FEMS Microbiol. Lett.* **106,** 39–46 (1993).
4. Paris, S. *et al.* Catalases of Aspergillus fumigatus. *Infect. Immun.* **71,** 3551–3562 (2003).
5. Calera, J. A. *et al.* Cloning and disruption of the antigenic catalase gene of Aspergillus fumigatus. *Infect. Immun.* **65,** 4718–4724 (1997).
6. Lamarre, C., Ibrahim-Granet, O., Du, C., Calderone, R. & Latgé, J.-P. Characterization of the SKN7 ortholog of Aspergillus fumigatus. *Fungal Genet. Biol. FG B* **44,** 682–690 (2007).
7. Hoi, J. W. S. *et al.* A novel family of dehydrin-like proteins is involved in stress response in the human fungal pathogen Aspergillus fumigatus. *Mol. Biol. Cell* **22,** 1896–1906 (2011).
8. Schrettl, M. *et al.* HapX-mediated adaption to iron starvation is crucial for virulence of Aspergillus fumigatus. *PLoS Pathog.* **6,** e1001124 (2010).
9. Schrettl, M. *et al.* SreA-mediated iron regulation in Aspergillus fumigatus. *Mol. Microbiol.* **70,** 27–43 (2008).
10. Schrettl, M. *et al.* Distinct roles for intra- and extracellular siderophores during Aspergillus fumigatus infection. *PLoS Pathog.* **3,** 1195–1207 (2007).

11. Schrettl, M. *et al.* Siderophore biosynthesis but not reductive iron assimilation is essential for Aspergillus fumigatus virulence. *J. Exp. Med.* **200,** 1213–1219 (2004).
